# The Functional Interaction Between PRDM16 and the SREBP Pathway Controls Lipid Metabolism

**DOI:** 10.3390/ijms262110246

**Published:** 2025-10-22

**Authors:** Hafiz Majid Mahmood, Maria Teresa Bengoechea-Alonso, Dana E. Al-Ansari, Khaled Machaca, Johan Ericsson

**Affiliations:** 1 College of Health and Life Sciences, Hamad Bin Khalifa University, Doha P.O. Box 34110, Qatar; 2 Calcium Signaling Group, Research Department, Weill Cornell Medicine Qatar, Doha P.O. Box 24144, Qatar; dea4012@qatar-med.cornell.edu (D.E.A.-A.); khm2002@qatar-med.cornell.edu (K.M.); 3 School of Medicine and Medical Science, University College Dublin, D04 C1P1 Dublin, Ireland

**Keywords:** SREBP, PRDM16, WAT, BAT, adipogenesis, lipid metabolism, cholesterol, diabetes, insulin signaling

## Abstract

Dysregulated lipid metabolism is associated with cardiovascular disease, obesity and type 2 diabetes. In the current report, we explore the functional interactions between two important regulators of lipid metabolism, sterol regulatory element-binding protein 1 and 2 (SREBP1/2), and PRDI-BF1 and RIZ homology domain containing 16 (PRDM16). The SREBP family of transcription factors regulate cholesterol and fatty acid synthesis and metabolism, primarily in liver but also in white adipose tissue. PRDM16 is a major regulator of brown adipose tissue (BAT) biogenesis and function as well as an inhibitor of white adipogenesis. We find that PRDM16 interacts with the nuclear forms of SREBP1/2 and inhibits their transcriptional activities. Consequently, inactivation of PRDM16 enhances the expression of well-established SREBP target genes involved in fatty acid and cholesterol synthesis/metabolism. Importantly, PRDM16 inactivation increases the expression of LDL receptor mRNA (1.6-fold) and protein (1.7-fold) and augments the cellular uptake of LDL particles (2.3-fold). Supporting these findings, PRDM16-deficient cells accumulate more neutral lipids in a SREBP1/2-dependent manner. Inactivation of PRDM16 in white and brown preadipocyte cell lines and human adipose-derived stem cells enhances the expression of SREBP target genes. In addition, the expression of adipogenic markers was increased in mature white adipocytes generated from PRDM16-knockdown preadipocytes (1.8- to 3.9-fold). Thus, our study identifies PRDM16 as a novel inhibitor of SREBP-dependent lipid metabolism with implications for adipose biology and metabolic disease.

## 1. Introduction

Dysregulated lipid metabolism is associated with several human diseases, including cardiovascular disease, obesity, and type 2 diabetes (T2D) [1,2,3]. The steady rise of these diseases has created a global cardiometabolic health crisis, with wide-ranging health, social, and economic consequences [4]. Whole-body lipid homeostasis relies on both dietary fat and de novo synthesis, primarily in the liver, to supply structural lipids and energy substrates [5]. White adipose tissue (WAT) stores triglycerides and releases fatty acids during fasting, whereas brown adipose tissue (BAT) oxidizes fatty acids to fuel thermogenesis [6,7]. The balance between lipid storage in WAT and lipid utilization in BAT is a central determinant of metabolic health. Lipid metabolism is coordinated by insulin signaling, which helps explain why diseases associated with dysfunctional insulin signaling, including obesity and T2D, are also characterized by dysregulated lipid metabolism [8,9,10].

Sterol regulatory element-binding proteins (SREBP1a, SREBP1c, and SREBP2) are transcription factors that control the expression of genes involved in cholesterol, fatty acid, and triglyceride synthesis and metabolism [11,12,13,14]. SREBP1c and SREBP2 are expressed in most mammalian cells and control genes involved in fatty acid/triglyceride and cholesterol synthesis, respectively. SREBP1a is the strongest transcription factor of the family and activates most SREBP target genes. Thus, SREBP1a is highly expressed in rapidly dividing cells, including cancer cells, to ensure a sufficient supply of lipids to support cell growth/proliferation. All three SREBPs are synthesized as large precursor proteins that are inserted into the endoplasmic reticulum (ER) membrane and need to be proteolytically cleaved to generate the active transcription factors [15,16,17]. The activation of SREBP1/2 is dependent on their transport from the ER to the Golgi, a process that is regulated by intracellular cholesterol levels and insulin signaling. In the ER, the SREBP precursor proteins interact with a sterol-sensing chaperone protein known as SREBP cleavage activating protein (SCAP). When mammalian cells are deprived of cholesterol, SCAP escorts SREBP1/2 in COPII vesicles from the ER to the Golgi. In the Golgi, two proteases (S1P and S2P) sequentially cleave SREBP1/2, releasing the N-terminal transcriptional domains, which translocate to the nucleus and activate genes involved in fatty acid and cholesterol synthesis and uptake [11]. When the amount of cholesterol in the ER membrane exceeds a certain threshold, cholesterol binds to SCAP, and this induces the SCAP/SREBP complex to bind to specific ER retention proteins, insulin-induced gene (Insig) 1/2. The SREBP/SCAP/Insig complex is retained in the ER, and the expression of SREBP target genes drop. The most important SREBP target gene from a clinical perspective is the LDL receptor gene [18]. This is illustrated by the fact that the SREBP-dependent induction of LDL receptor mRNA and protein in the liver is responsible for the LDL-cholesterol lowering activity of the statin family of drugs [19], the most prescribed cholesterol-lowering drug globally. SREBP1c expression and activation are induced in response to insulin signaling [20,21].

Transcriptionally active SREBP1/2 molecules are unstable and degraded by the ubiquitin-proteasome pathway in a phosphorylation-dependent manner [22,23,24,25]. Interestingly, the phosphorylation-dependent degradation of nuclear SREBP1/2 is negatively regulated by insulin signaling. We have proposed that the rapid degradation of transcriptionally active SREBP1/2 is part of a negative feedback loop that forces cells to re-sense their intracellular (cholesterol) and/or extracellular (insulin) environment before activating additional precursor proteins [26].

The mRNA expression of SREBP1a and SREBP1c are induced during white adipogenesis in vitro [27,28,29]. The levels of SREBP1c mRNA and SREBP1 protein are also high in white adipose tissue (WAT). It has been shown that SREBP1c promotes/supports white adipogenesis in vitro. This involves the SREBP-dependent activation of the PPARγ and fatty acid synthase genes, with the latter driving fatty acid synthesis and thereby the generation of PPARγ ligands/activators [28,30]. However, whether SREBP1c has a similar role in white adipogenesis in vivo is unclear [31]. Nevertheless, it is well established that SREBP1c plays important roles in maintaining lipid metabolism in mature WAT in vivo, and that the activation of SREBP1c in this tissue can be modulated in obesity [27,32,33,34,35,36].

The functional role of SREBP1/2 in brown adipocytes has not been explored extensively. However, it has been reported that the expression and activation of SREBP1c are increased in BAT in response to chronic cold exposure [37]. As a result, the expression of SREBP1c target genes involved in fatty acid synthesis was also induced under these conditions. Importantly, inactivating SREBP1/2 in BAT reduced the thermogenic capacity of mice in response to chronic cold. The authors concluded that SREBP1c-dependent fatty acid synthesis is induced in BAT to supply fatty acids to meet the increased demand for fatty acid β-oxidation and heat generation in response to long-term cold exposure, suggesting that SREBP1c has a functional role in mature brown adipocytes.

White adipocytes are characterized by the accumulation of a single triglyceride-rich lipid droplet that takes up most of the intracellular space. The triglycerides in this droplet are hydrolyzed during fasting, and the fatty acids enter the circulation to provide energy for other cells and tissues. Brown adipocytes contain several smaller lipid droplets and are characterized by their high mitochondrial content. The lipid droplets found in brown adipocytes are also hydrolyzed, and the liberated fatty acids are transported to mitochondria and converted to acetyl-CoA through β-oxidation. The acetyl-CoA feeds into the Krebs cycle and electron transport chain (ETC). Brown adipocytes express a specific uncoupling protein, UCP1, which dissipates the proton gradient created by the ETC in a manner independent of ATP synthesis. Instead, the energy contained in the gradient is converted to heat. Although white and brown adipocytes share many characteristics, their developmental origins are distinct [38,39]. Despite this, white and brown adipogenesis share many regulatory proteins, including PPARγ and members of the C/EBP family of transcription factors. A third cell type, beige adipocytes, are found within WAT and can adopt a more brown phenotype in response to various external signals, such as cold, exercise, and beta-adrenergic agonists [40,41]. This process, known as WAT browning, enhances thermogenic capacity and increases organismal energy consumption.

The transcription factor PPARγ is known as a master regulator of white adipogenesis [42,43]. The differentiation of white progenitor cells (preadipocytes) is a transcriptional cascade involving PPARγ and members of the C/EBP family of transcription factors. Together, PPARγ and C/EBPα/β/δ activate the expression of multiple genes involved in adipocyte differentiation, including additional transcription factors and genes involved in lipid synthesis, and lipid and glucose uptake. This process is activated in response to insulin signaling, and fully mature adipocytes are very insulin responsive [44,45]. PRDI-BF1 and RIZ homology domain containing 16 (PRDM16) is a transcriptional coregulator that promotes the expression of brown/beige-specific genes and suppresses myocyte- and WAT-specific genes [46,47,48]. PRDM16 accomplishes this by interacting with other transcriptional regulators. The most important of these include PPARγ, C/EBPβ, PGC1α, CtBP1/2, and EHMT1 [47,48,49,50,51,52]. The expression of PRDM16 and C/EBPβ is sufficient to drive brown adipogenesis in fibroblasts and myoblasts, and the activation of PGC1α and PPARα is important for mitochondrial biogenesis and for establishing the thermogenic program. Notably, PRDM16 function in WAT appears critical for overall thermogenic potential and energy expenditure [41,51]. This was elegantly illustrated using a mouse model in which PRDM16 was inactivated after the brown fat/muscle fate decision was established [40]. Although these mice presented no obvious BAT phenotype, they displayed a dramatic reduction in WAT browning, most likely linked to blunted beige adipogenesis and/or function. The PRDM16-deficient animals developed obesity earlier than control animals in response to high-fat feeding and displayed severe insulin resistance and hepatic steatosis, even before any obvious weight gain [40]. Thus, PRDM16, beige adipocytes, and the browning of WAT are critical for whole-body metabolic health as they promote thermogenesis and energy consumption.

In this investigation, we set out to explore if there was any functional interaction(s) between SREBP1/2 and PRDM16. We demonstrate that PRDM16, the master regulator of brown adipogenesis, physically interacts with nuclear SREBP1/2. Using loss- and gain-of-function assays, we demonstrate that PRDM16 inhibits the transcriptional activities of SREBP1/2. As a result, inactivation of PRDM16 activates the expression of SREBP target genes involved in lipid synthesis and metabolism, resulting in the accumulation of intracellular lipid droplets. Importantly, we demonstrate that the functional interaction between PRDM16 and SREBP1/2 extend to progenitors of both white and brown adipocytes, suggesting that this regulatory axis could affect adipocyte differentiation and/or function.

## 2. Results

### 2.1. PRDM16 Represses SREBP Target Promoters in an SREBP-Dependent Manner

**Figure 1:**
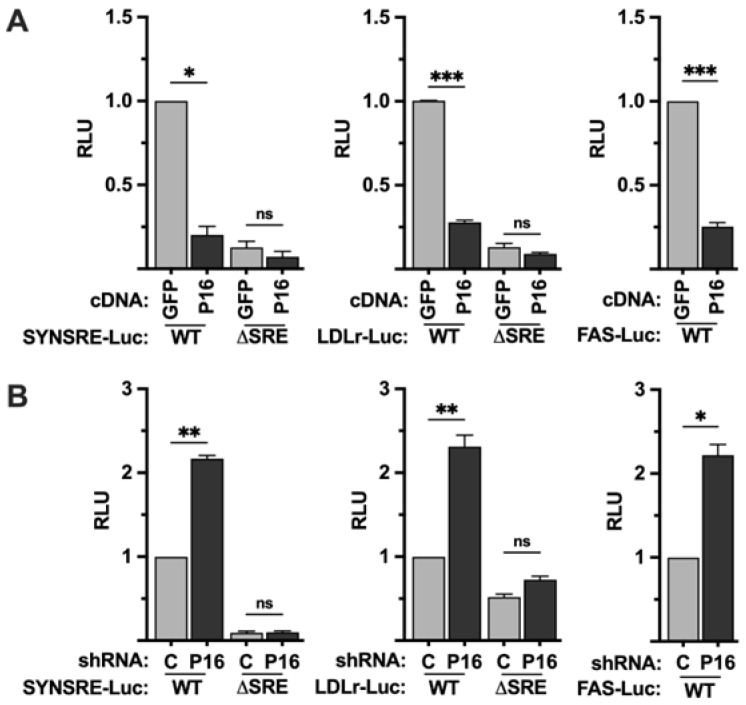
PRDM16 represses SREBP target gene promoters. (**A**) HepG2 cells were transfected with HMG-CoA synthase (SYNSRE), LDL receptor (LDLR), or fatty acid synthase (FAS) promoter-luciferase constructs together with either GFP or PRDM16 cDNA. In the case of HMG-CoA synthase and the LDL receptor promoter-reporter constructs, two constructs were used, either wild-type (WT) or a version in which the SREBP binding site was deleted *(*ΔSRE). Forty-eight hours after transfection, the cells were lysed, and luciferase activity was measured. (**B**) HepG2 cells were transfected with the promoter-reporter constructs mentioned in (**A**) together with non-targeted (C) or PRDM16 (P16) shRNA. Forty-eight hours after transfection, the cells were lysed, and luciferase activity was measured. Cells were also transfected with the β-galactosidase gene as an internal control for transfection efficiency. Luciferase values (relative light units, RLU) were calculated by dividing the luciferase activity by the β-galactosidase activity. The data represent the average −/+ SEM of at least three independent experiments performed in duplicates. The RLU of WT promoter-reporter constructs transfected with either GFP or non-targeted shRNA were set to 1. *p*-values lower than 0.05 were considered statistically significant. * *p* < 0.05, ** *p* < 0.01, and *** *p* < 0.001. ns, not significant.

In order to explore if PRDM16 affects SREBP-dependent transcription, we used promoter-reporter constructs containing the promoters of well-established SREBP target genes, i.e., HMG-CoA synthase (SYNSRE), the LDL receptor (LDLR), and fatty acid synthase (FAS), in promoter-reporter assays. These promoter-reporters were transfected into HepG2 cells together with plasmids expressing an unrelated cDNA (GFP) or PRDM16 cDNA. As seen in Figure 1A, the expression of PRDM16 suppressed the activity of all three promoter-reporter genes. Conversely, the activities of the same promoter-reporters were enhanced in cells expressing PRDM16-targeted shRNA (Figure 1B). Although these promoters are regulated by SREBP1/2, they also contain binding sites for other transcription factors. To determine if the PRDM16-mediated regulation of the HMG-CoA synthase and LDL receptor promoters was dependent on SREBP1/2, the same gain- and loss-of-function assays were performed with the corresponding promoter-reporter constructs in which the SREBP binding sites had been deleted (ΔSRE). As seen in Figure 1, deletion of the SREBP binding sites in these promoters made them insensitive to PRDM16, suggesting that PRDM16 interferes with the transcriptional activities of SREBP1/2. The basal expression and knockdown efficiency of PRDM16 protein in HepG2 cells are illustrated in Figure S4A.

### 2.2. PRDM16 Is Unable to Regulate the Transcriptional Activities of SREBP1/2 Under Repressive Conditions

**Figure 2:**
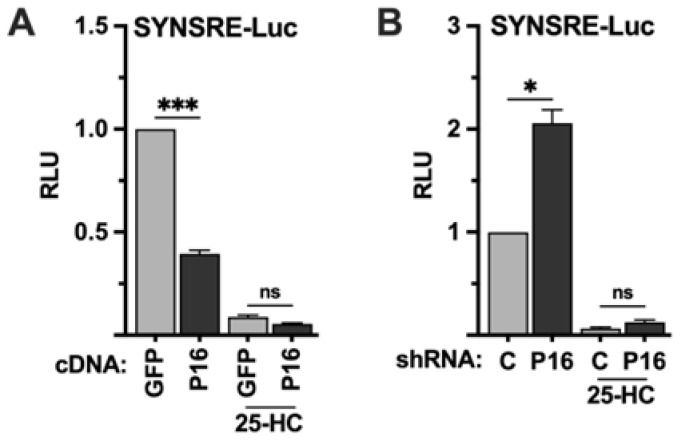
The PRDM16-mediated repression of SREBP target promoters is dependent on SREBP1/2 activation. (**A**) HepG2 cells were transfected with the HMG-CoA synthase promoter-luciferase construct (SYNSRE) together with GFP or PRDM16 (P16) cDNA. Twenty-four hours after transfection, the cells were placed in lipoprotein-deficient media. Where indicated, media was supplemented with 25-hydroxycholesterol (25-HC) to block SREBP1/2 activation. Forty-eight hours after transfection, the cells were lysed, and luciferase activity was measured. (**B**) HepG2 cells were transfected with the HMG-CoA synthase promoter-luciferase construct (SYNSRE) together with non-targeted (C) or PRDM16 (P16) shRNA and treated as in (**A**). Cells were also transfected with the β-galactosidase gene as an internal control for transfection efficiency. Luciferase values (relative light units, RLU) were calculated by dividing the luciferase activity by the β-galactosidase activity. The data represent the average −/+ SEM of at least three independent experiments performed in duplicate. The RLU of WT promoter-reporter constructs transfected with either GFP or non-targeted shRNA were set to 1. *p*-values lower than 0.05 were considered statistically significant. * *p* < 0.05, and *** *p* < 0.001. ns, not significant.

The previous set of experiments indicated that PRDM16 is a negative regulator of SREBP target promoters, and that this regulation is dependent on the SREBP binding sites in these promoters. The activation of SREBP1/2 is negatively regulated by the cellular accumulation of cholesterol and/or cholesterol metabolites, including 25-hydroxycholesterol (25-HC). To test if the PRDM16-mediated regulation of the HMG-CoA synthase promoter was dependent on the activation of SREBP1/2, HepG2 cells were transfected with the SYNSRE promoter-reporter and either GFP or PRDM16 cDNA and left in lipoprotein-depleted media (LDM) or the same media supplemented with 25-HC. As seen in Figure 2A, PRDM16 attenuated the expression of the reporter gene in LDM but was unable to do so in the presence of 25-HC. Conversely, shRNA-mediated inactivation of PRDM16 enhanced the expression of the reporter gene in cells grown in LDM but was unable to do so in the presence of 25-HC (Figure 2B), suggesting that the repressive effect of PRDM16 is dependent on the activation of endogenous SREBP1/2.

### 2.3. PRDM16 Targets the Nuclear Forms of SREBP1/2

**Figure 3:**
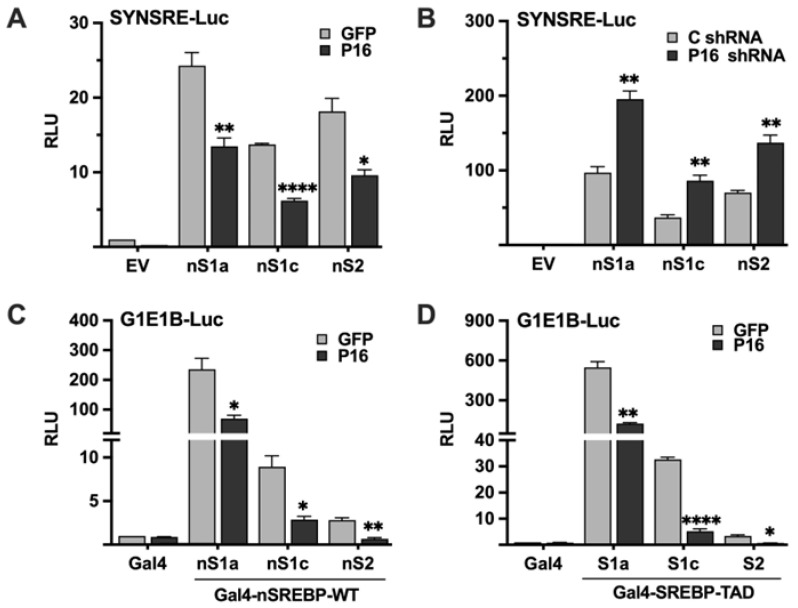
PRDM16 targets the nuclear forms of SREBP1a, SREBP1c, and SREBP2. (**A**) HepG2 cells were transfected with the HMG-CoA synthase promoter-luciferase construct (SYNSRE) together with GFP or PRDM16 (P16) cDNA in the absence (EV) or presence of cDNA encoding the nuclear forms of SREBP1a (nS1a), SREBP1c (nS1c), or SREBP2 (nS2). Forty-eight hours after transfection, cells were lysed, and luciferase activity was measured. (**B**) HepG2 cells were transfected with the HMG-CoA synthase promoter-luciferase construct (SYNSRE) together with non-targeted (C) or PRDM16 (P16) shRNA in the absence (EV) or presence of cDNA encoding the nuclear forms of SREBP1a (nS1a), SREBP1c (nS1c), or SREBP2 (nS2). Forty-eight hours after transfection, cells were lysed, and luciferase activity was measured. EV, empty vector. (**C**) HepG2 cells were transfected with a minimal promoter-luciferase construct containing a single binding site for the yeast transcription factor Gal4 (G1E1B-Luc) together with expression vectors for the DNA binding domain of Gal4 (Gal4), or the same DNA binding domain fused to the nuclear forms of SREBP1a (nS1a), SREBP1c (nS1c), or SREBP2 (nS2), and either GFP or PRDM16 (P16). Forty-eight hours after transfection, cells were lysed, and luciferase activity was measured. (**D**). HepG2 cells were transfected with the same promoter-reporter construct as in (**A**) together with the DNA binding domain of Gal4 (Gal4), or the same DNA binding domain fused to the transactivation domain (TAD) of SREBP1a (nS1a), SREBP1c (nS1c), or SREBP2 (nS2), and either GFP or PRDM16 (P16) cDNA. Forty-eight hours after transfection, cells were lysed, and luciferase activity was measured. Cells were also transfected with the β-galactosidase gene as an internal control for transfection efficiency. Luciferase values (relative light units, RLU) were calculated by dividing the luciferase activity by the β-galactosidase activity. The data represent the average −/+ SEM of at least three independent experiments performed in duplicates. The RLU of WT promoter-reporter constructs transfected with either GFP or non-targeted shRNA were set to 1. *p*-values lower than 0.05 were considered statistically significant. * *p* < 0.05, ** *p* < 0.01, and **** *p* < 0.0001.

The experiments above demonstrated that PRDM16 represses SREBP target promoters in an SREBP-dependent manner, suggesting that it targets active SREBP1/2 molecules. To test this hypothesis, HepG2 cells were transfected with the SYNSRE promoter-reporter and the nuclear forms of SREBP1a, SREBP1c, and SREBP2, and either GFP or PRDM16 cDNA. As seen in Figure 3A, PRDM16 inhibited the transcriptional activities of all three forms of SREBPs. The opposite result was observed when the experiment was repeated with untargeted or PRDM16 shRNA; inactivation of PRDM16 enhanced the activities of the ectopically expressed SREBPs (Figure 3B). Taken together, these results suggest that PRDM16 can repress the transcriptional activities of active SREBP molecules. To explore this possibility further, we took advantage of the yeast Gal4 system. In this system, the expression of the reporter gene is dependent on a single binding site for the yeast transcription factor Gal4. Thus, the nuclear SREBPs need to be fused to the DNA-binding domain of Gal4 to be able to bind and activate the promoter-reporter. Thus, the transcriptional activities of SREBP1/2 are uncoupled from their intrinsic DNA-binding activities in this system. Gal4 fusions of all three SREBPs activated the reporter gene and were repressed by coexpressed PRDM16 (Figure 3C) This experiment was repeated with Gal4 fusions containing only the transactivation domains (TADs) of the three SREBPs. Again, all three TADs were able to activate the reporter gene and were repressed by coexpressed PRDM16 (Figure 3D). Taken together, these experiments demonstrate that PRDM16 represses the nuclear forms of SREBP1a, SREBP1c and SREBP2 in a manner that is independent of the DNA-binding domain of these proteins, and that ectopically expressed PRDM16 can target the transactivation domains of SREBP1/2. The basal expression and knockdown efficiency of PRDM16 protein in HepG2 cells are illustrated in Figure S4A.

### 2.4. PRDM16 Interacts with Nuclear SREBP1/2

**Figure 4:**
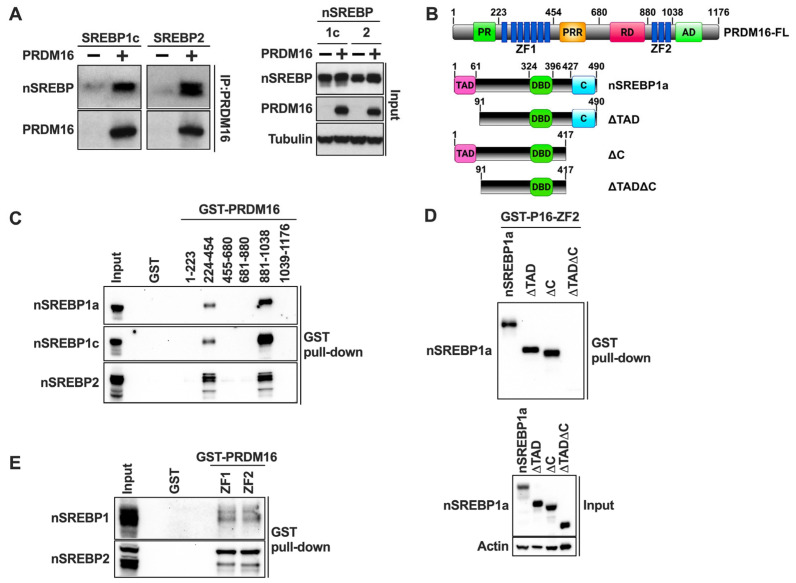
PRDM16 interacts with nuclear SREBP1/2 through its zinc finger domains. (**A**) HEK 293 cells were transfected with cDNA encoding the nuclear forms of either SREBP1c or SREBP2 in the absence (−) or presence (+) of HA-tagged PRDM16. Forty-eight hours after transfection, cells were lysed, precleared, and PRDM16 was immunoprecipitated with anti-HA antibodies. The immunoprecipitated proteins were resolved on SDS-PAGE gels and transferred to nitrocellulose membranes. The amount of nuclear SREBP1c and SREBP2, and PRDM16 in the immunoprecipitated material was determined by Western blotting (left). The levels of nuclear SREBP1c and SREBP2, PRDM16, and α-tubulin (loading control) in the whole cell lysates (Input) was determined by Western blotting (right). (**B**) A schematic structure of PRDM16 (top) with the numbering corresponding to the fragments used for GST pulldown assays, and a schematic structure of nuclear SREBP1a and the different deletion mutants used in the GST pulldown assays (bottom). (**C**) HEK293 cells were transfected with expression vectors for the nuclear forms of SREBP1a, SREBP1c, or SREBP2, and whole-cell lysates were used in GST pulldown experiments with the indicated PRDM16 fragments. The captured proteins were separated on SDS-PAGE gels, and the amount of the individual nuclear SREBP (nSREBP) proteins were analyzed by Western blotting. GST alone was used as a negative control in the pulldown experiments and was run out on the same gel together with the whole-cell lysates (Input). The Coomassie staining of the PRDM16 GST fusion proteins is shown in Figure S1A. (**D**) HEK293 cells were transfected with expression vectors encoding full-length nuclear SREBP1a (nSREBP1a), or the mutants illustrated in (**B**). These mutants contained deletions of the N-terminal transactivation domain (ΔTAD), the C-terminal regulatory domain (ΔC), or both domains (ΔTADΔC). Whole-cell lysates were used in GST pulldown assays using GST-ZF2 as bait. The pull-down material (top) and whole-cell lysates (Input, bottom) were analyzed by Western blotting. The Coomassie staining of the GST-ZF1 and GST-ZF2 proteins is shown in Figure S1B. (**E**) Nuclear extracts were prepared from MCF7 cells grown in lipoprotein-deficient media to fully activate SREBP1/2. The nuclear extracts were used in GST pulldown assays using GST-ZF1 and ZF2 of PRDM16, with GST alone as negative control. The captured material was separated on SDS-PAGE gels together with an aliquot of the nuclear extracts used in the pulldown assays (Input) and analyzed by Western blotting. The Coomassie staining of GST-ZF1 and ZF2 is shown in Figure S1B.

The previous experiments demonstrated that PRDM16 is a negative regulator of the transcriptional activities of nuclear SREBP1/2, suggesting that the two proteins could interact. To explore this possibility, we performed traditional co-immunoprecipitation experiments using HEK293 cells expressing either nuclear SREBP1c or SREBP2 in the absence or presence of HA-tagged PRDM16. Following cell lysis, PRDM16 was immunoprecipitated with anti-HA antibodies and the precipitated proteins separated on SDS-PAGE gels. As illustrated in Figure 4A, both SREBP1c and SREBP2 were found to interact with PRDM16.

### 2.5. PRDM16 Interacts with Nuclear SREBP1/2 Through Its Zinc Finger Domains

In an effort to identify the SREBP-interacting domain(s) in PRDM16, we purified several GST fusion proteins containing different PRDM16 domains from bacteria (Figure 4B, top). The purified proteins were used in GST pulldown assays together with lysates obtained from HEK293T cells expressing either nuclear SREBP1a, SREBP1c, or SREBP2. As seen in Figure 4C, all three SREBPs were pulled down with GST fusions containing either of the two zinc fingers in PRDM16 (ZF1 or ZF2), suggesting that these domains are important for the PRDM16-SREBP1/2 interaction. The levels and purity of the GST-PRDM16 fragments used in these assays are illustrated in Figure S1A.

To identify the PRDM16-interaction domains in SREBP1, GST-ZF1 and GST-ZF2 were used in GST pulldown assays with lysates from HEK293 cells expressing nuclear SREBP1a, either wild-type or specific deletion mutants targeting the N-terminal transactivation domain (ΔTAD), the C-terminal regulatory domain (ΔC), or both domains (ΔTADΔC) (Figure 4B, bottom). Although deletion of these domains alone failed to reduce the interaction with ZF2 in PRDM16, the simultaneous deletion of both domains eliminated the interaction (Figure 4D), suggesting that both the N-terminal TAD and the C-terminal regulatory domain in nuclear SREBP1 are required for its interaction with PRDM16. The same results were obtained with ZF1 (Figure S1B). The levels and purity of the GST-ZF1 and ZF2 used in these assays are illustrated in Figure S1B. These results are in line with our observation that PRDM16 can repress the transcriptional activities of the TADs of SREBP1a, SREBP1c, and SREBP2 (Figure 3D).

The previous experiments were based on the ectopic expression of nuclear SREBP1/2. In order to determine if the two ZFs in PRDM16 could interact with endogenous nuclear SREBP1/2, we used cell lysates from MCF7 cells grown in lipoprotein-deficient media in our GST pulldown assays. As seen in Figure 4E, both zinc fingers interacted with endogenous nuclear SREBP1 and SREBP2. Unfortunately, the protein expression of PRDM16 was very low in all cell lines tested, which prevented us from exploring whether endogenous PRDM16 and nuclear SREBP1/2 interact.

### 2.6. PRDM16 Interacts with SREBP1 Bound to Target Promoters In Vitro

**Figure 5:**
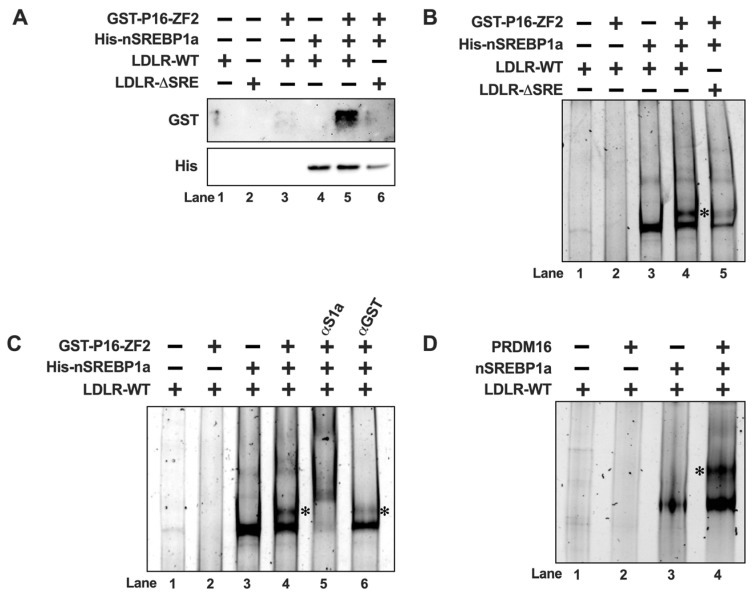
PRDM16 is recruited to SREBP target promoters through its interaction with SREBP1/2. (**A**) GST-tagged PRDM16-ZF2 (GST-P16-ZF2) and 6xHis-tagged nuclear SREBP1a (His-nSREBP1a) were used in DNA-precipitation assays using two different biotin-labeled LDL receptor promoter probes, either wild-type (LDLR-WT) or the corresponding SREBP-binding site deletion (LDLR-ΔSRE). The recombinant proteins were mixed with the promoter probes, either individually or together, and the DNA-protein complexes were captured on streptavidin-coated magnetic beads. Following extensive washing, the captured proteins were separated on SDS-PAGE gels, and the amounts of ZF2 (GST) and nSREBP1a (His) were analyzed by Western blotting. (**B**) GST-P16-ZF2 and 6xhis-nSREBP1a were mixed with the LDLR-WT or LDLR-ΔSRE unlabeled promoter probes and the DNA-protein complexes were resolved on native PAGE gels. DNA-protein complexes were visualized with SYBR Safe. The ZF2-SREBP1a-DNA complex is indicated by an asterisk (*). (**C**) GST-P16-ZF2 and 6xhis-nSREBP1a were mixed with the LDLR-WT unlabeled promoter probes. Prior to loading the samples on native PAGE gels, either anti-GST (ZF2, αGST) or anti-His (nSREBP1a, αS1a) were added to the mixtures as indicated. DNA-protein complexes were visualized with SYBR Safe. The ZF2-SREBP1a-DNA complex is indicated by an asterisk (*). (**D**) HEK293 cells were transfected with expression vectors for nuclear SREBP1a (nSREBP1a) or PRDM16. Forty-eight hours after transfection, nuclear extracts were prepared and incubated with an unlabeled LDLR promoter probe (LDLR-WT). The reaction mixtures were resolved on native PAGE gels, and the DNA-protein complexes were visualized with SYBR Safe. The PRDM16-SREBP1a-DNA complex is indicated by an asterisk (*).

The previous data suggest that PRDM16 interacts with nuclear SREBP1/2, and that this interaction is sufficient for their PRDM16-mediated inactivation, suggesting that PRDM16 could be recruited to SREBP target promoters. To test this hypothesis, we initially used recombinant nuclear SREBP1a and ZF2 of PRDM16 in DNA precipitation assays (DNAPs). In these assays, a biotin-labelled DNA probe corresponding to the cholesterol-responsive portion of the LDL receptor (LDLR) promoter was mixed with ZF2 in the absence or presence of recombinant SREBP1a. The biotin-labelled DNA was captured on streptavidin-coated beads, followed by extensive washing and separation of DNA-associated proteins on SDS-PAGE gels. As illustrated in Figure 5A, there was no binding of ZF2 to the DNA probe in the absence of SREBP1a (lane 3). However, the amount of ZF2 associated with the DNA probe was greatly enhanced in the presence of nuclear SREBP1a (lane 5), suggesting that PRDM16 is recruited to DNA through its interaction with nuclear SREBP molecules. This hypothesis was strengthened when an LDLR promoter probe with a deleted SREBP binding site (LDLRΔSRE) was used in the DNAP. ZF2 failed to interact with the ΔSRE probe even in the presence of nuclear SREBP1a (lane 6 in Figure 5A). Similar results were obtained for ZF1 (Figure S2A). The levels and purity of GST-ZF1 and ZF2 are illustrated in Figure S2B. Since these assays used recombinant proteins, the interaction between nuclear SREBP1a and PRDM16 is most likely direct. The LDLR promoter probes used in these experiments are identical to those used in the promoter-reporter assays experiments illustrated in Figure 1.

Although unlikely in this case, it is possible that proteins could be captured by the streptavidin beads independently of the biotin-labelled DNA probes. To avoid this possibility, we decided to monitor the recruitment of ZF2 to the LDLR promoter in electromobility shift assays (EMSAs). As expected, recombinant SREBP1a interacted with the probe, resulting in a single protein-DNA complex in the native PAGE gel (Figure 5B, lane 3). In agreement with the DNAP assays, ZF2 failed to interact with the probe in the absence of SREBP1a (lane 2). When both proteins were added at the same time, we detected a second, larger, protein-DNA complex (lane 4). Both complexes were reduced when using the ΔSRE LDLR probe (lane 5), suggesting that the ZF2-specific complex is dependent on the initial formation of the SREBP1a-specific complex. Similar results were observed for ZF1 (Figure S3). To further explore the composition of the two complexes observed in the presence of both SREBP1a and ZF2, we repeated the assays above and introduced antibodies directed against either SREBP1 or ZF2 (α-GST) in the assays. As seen earlier, addition of both SREBP1a and ZF2 to the reaction resulted in two distinct protein-DNA complexes. Both complexes were further shifted upwards following the addition of SREBP1 antibodies to the reaction (Figure 5C, lane 5), suggesting that both complexes contain SREBP1a. However, rather than shifting any of the two complexes, the GST antibody specifically diminished the larger complex (lane 6), suggesting that this complex contains GST-ZF2. Although it is not unusual for the addition of antibodies to EMSA reactions to interfere with the formation of specific protein-DNA complexes, we cannot exclude the possibility that the antibody-ZF2-SREBP1a-DNA complex ended up being very large, and therefore not resolved in the native gel. Taken together, our results suggest that PRDM16 is recruited to SREBP target promoters through a direct interaction with nuclear SREBP1/2, at least in vitro. This hypothesis was reinforced when we used nuclear extracts from HEK293 cells expressing nuclear SREBP1a or full-length PRDM16 in the EMSA assay. As seen in Figure 5D, PRDM16 only bound to the DNA probe in the presence of SREBP1a. Unfortunately, the low expression of PRDM16 and the low specificity of available PRDM16 antibodies prevented us from exploring if endogenous PRDM16 is recruited to SREBP target promoters in vivo. However, this is an important question that should be explored in future work.

### 2.7. Loss of PRDM16 Results in the Induction of SREBP Target Genes in an SREBP-Dependent Manner

**Figure 6:**
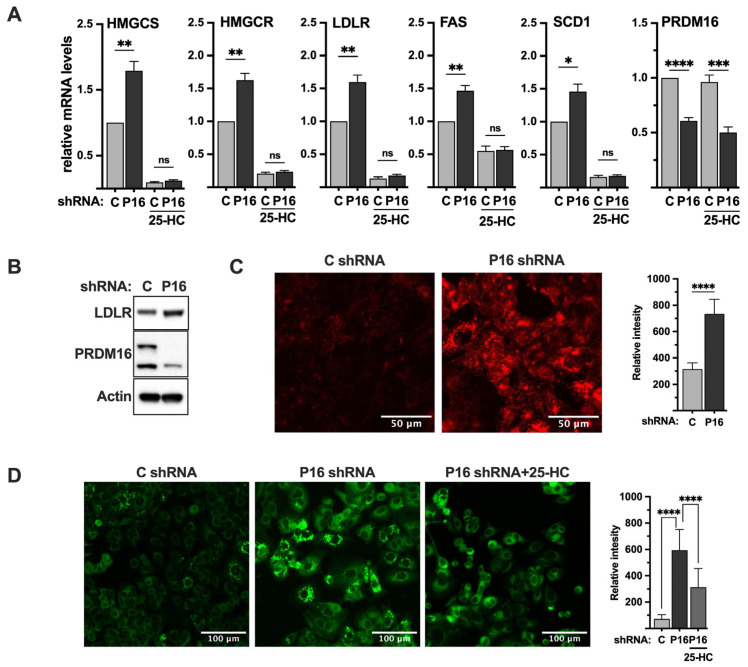
PRDM16 represses SREBP target gene expression and SREBP-dependent lipid metabolism. (**A**) MCF7 cells were transduced with non-targeted (C) or PRDM16 (P16) shRNA. Seventy-two hours after transduction, the media was changed to lipoprotein-deficient media, which was supplemented with 25-hydroxycholesterol (25-HC) where indicated. Ninety-six hours after transduction, mRNA was extracted and used for qPCR with primers specific for HMG-CoA synthase (HMGCS), HMG-CoA reductase (HMGCR), the LDL receptor (LDLR), fatty acid synthase (FAS), stearoyl-CoA desaturase (SCD1), and PRDM16. The relative mRNA expression in cells transduced with non-targeted shRNA and grown in the absence of 25-HC was set to 1. The data represent the average −/+ SEM of at least three independent experiments. (**B**) MCF7 cells were transduced with non-targeted (C) or PRDM16 (P16) shRNA. Seventy-two hours after transduction, whole-cell lysates were prepared and separated on SDS-PAGE gels. The amount of LDL receptor, PRDM16, and actin (loading control) was analyzed by Western blotting. (**C**) MCF7 cells were transduced as in (**B**). Seventy-two hours after transduction, the media was changed to lipoprotein-deficient media. Ninety-six hours after transduction, pHrodo-labeled LDL (red) was added to cells for 4 h, followed by repeated washes with PBS containing BSA (0.3%). The mean fluorescence intensities −/+ SD across each experimental group are provided in the bar graph (right). (**D**) MCF7 cells were transduced as in (**A**). Seventy-two hours after transduction, the media was changed to lipoprotein-deficient media alone or supplemented with 25-hydroxycholesterol (25-HC). Ninety-six hours after transduction, cells were fixed and stained with LipidTOX neutral lipid stain (green). The mean fluorescence intensities −/+ SD across each experimental group are provided in the bar graph (right). *p*-values lower than 0.05 were considered statistically significant. * *p* < 0.05, ** *p* < 0.01, *** *p* < 0.001, and **** *p* < 0.0001. ns, not significant.

Thus far, our data suggest that PRDM16 interacts with nuclear SREBP1/2, which enables its recruitment to SREBP target promoters, resulting in the repression of the transcriptional activities of SREBP1/2, at least in the context of SREBP-dependent promoter-reporter constructs. To explore if this is also the case for endogenous SREBP target genes, we used lentiviral delivery of PRDM16 shRNA in MCF7 cells and monitored the mRNA expression of HMG-CoA reductase, HMG-CoA synthase, fatty acid synthase, and the LDL receptor in the knockdown cells. We selected MCF7 cells for these experiments since they were found to express higher levels of PRDM16 compared to HepG2 cells, enabling us to monitor the knockdown efficiency at the mRNA (Figure 6A) and protein levels (Figure S4B). As illustrated in Figure 6A, shRNA-mediated inactivation of PRDM16 increased the expression of all five SREBP target genes in cells grown in lipoprotein-deficient media (inducing conditions). The induction of these genes was dependent on nuclear SREBP1/2 since inactivation of PRDM16 failed to enhance the expression of any of the genes in cells treated with 25-HC. Similar results were seen when the expression of HMG-CoA synthase and SCD1 protein was analyzed in control and PRDM16 knockdown MCF7 cells (Figure S5).

### 2.8. Loss of PRDM16 Results in the Induction of LDL Receptor Protein, LDL Uptake and Intracellular Lipid Accumulation

The LDL receptor gene is a clinically important SREBP target gene because of its link to cardiovascular disease and the function of statins. As seen in Figure 6A, inactivation of PRDM16 in MCF7 cells resulted in the induction of LDL receptor mRNA, resulting in increased levels of LDL receptor protein (Figure 6B). To determine if this increase in LDL receptor protein resulted in the enhanced uptake of LDL particles in PRDM16 knockdown cells, we used LDL labelled with a pH sensitive probe (red). This probe only becomes fluorescent once it enters cells, in this case through endocytosis, and is exposed to the acidic intracellular environment. As seen in Figure 6C, the uptake of labelled LDL particles was significantly enhanced in cells transduced with PRDM16 shRNA compared to cells transduced with untargeted control shRNA, suggesting that the PRDM16-dependent repression of SREBP1/2 affects receptor-mediated uptake of LDL.

Our data suggest that the inactivation of PRDM16 in MCF7 cells induces the expression of SREBP target genes involved in cholesterol and fatty acid synthesis, as well as the uptake of LDL particles. One possible outcome of these processes could be the accumulation of intracellular lipids. To explore this possibility, we stained MCF7 cells transduced with control or PRDM16 shRNA with a neutral lipid stain (green) to monitor the accumulation of intracellular lipids. As illustrated in Figure 6D, knockdown of PRDM16 resulted in enhanced accumulation of neutral lipids, especially in the form of lipid droplets. As seen in Figure 6A, the expression of the LDL receptor gene in MCF7 cells was significantly reduced in response to 25-HC. Thus, if the accumulation of lipid droplets in PRDM16 knockdown MCF7 cells was dependent on the LDL receptor, treating cells with 25-HC should reduce the accumulation of lipids. As seen in Figure 6D, the amount of intracellular lipids in the PRDM16 knockdown MCF7 cells was significantly reduced in cells treated with 25-HC, suggesting that the accumulation of lipids in PRDM16 knockdown cells is dependent on nuclear SREBP1/2.

PRDM16 is considered the master regulator of brown and beige adipogenesis, in part by controlling the expression of UCP1 and PGC1α. In addition, PRDM16 inhibits white adipogenesis. Thus, PRDM16 is a potent regulator of both brown and white adipose biology. At the same time, SREBP1c has been shown to play an important role during the differentiation of 3T3-L1 preadipocytes. To explore whether PRDM16 also controls SREBP1/2 in adipocyte cell models, we performed PRDM16 gain- and loss-of-function experiments in mouse 3T3-L1 (white) and WT-1 (brown) preadipocyte cell lines, as well as human adipose-derived stem cells (hADSCs), and monitored the expression of SREBP target genes and the impact on the differentiation of 3T3-L1 cells.

### 2.9. Ectopic Expression of PRDM16 Blocks the Differentiation of 3T3-L1 Cells

**Figure 7:**
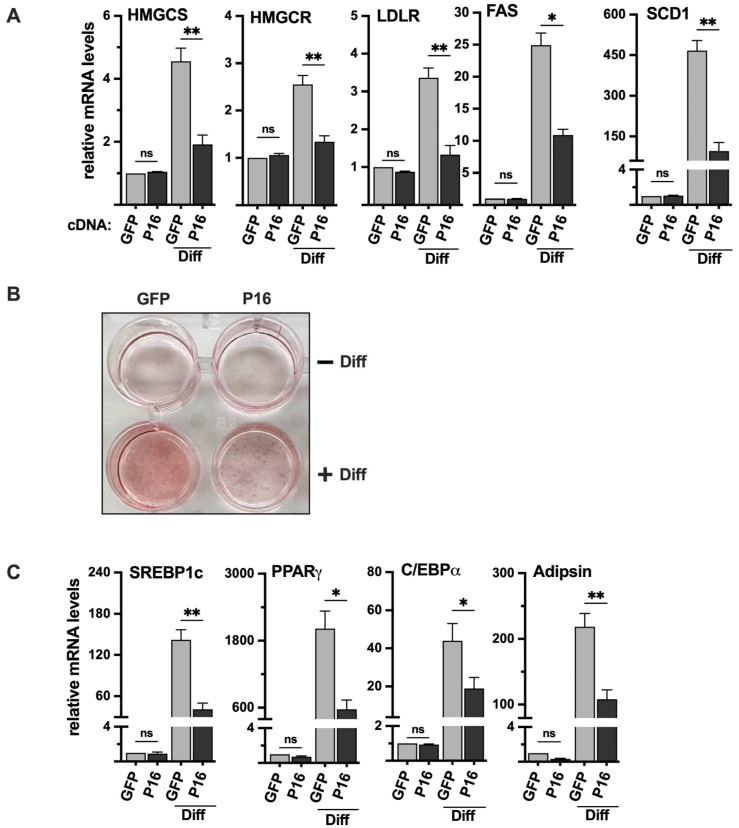
Ectopic expression of PRDM16 attenuates adipogenesis. (**A**) 3T3-L1 preadipocytes were transduced with expression vectors for GFP or PRDM16 (P16) and left untreated or differentiated to mature adipocytes (Diff). RNA was extracted, and the expression of HMG-CoA synthase (HMGCS), HMG-CoA reductase (HMGCR), the LDL receptor (LDLR), fatty acid synthase (FAS), and stearoyl-CoA desaturase (SCD1) was analyzed by qPCR. The relative expression of each gene in undifferentiated GFP-expressing cells was set to 1. The data represent the average −/+ SEM of at least three independent experiments (**B**) 3T3-L1 cells were transduced and treated as in (**A**), fixed and stained with oil red O to visualize the accumulation of lipids. The wells in the upper row are undifferentiated (−Diff), and those in the lower row are differentiated (+Diff). (**C**) 3T3-L1 cells were transduced and treated as in (**A**). RNA was extracted, and the expression of PPARγ, C/EBPα, adipsin, and SREBP1c in undifferentiated and differentiated (Diff) cells was analyzed by qPCR. The relative expression of each gene in undifferentiated GFP-expressing cells was set to 1. The data represent the average −/+ SEM of at least three independent experiments. *p*-values lower than 0.05 were considered statistically significant. * *p* < 0.05, and ** *p* < 0.01. ns, not significant.

Initially, we transduced 3T3-L1 preadipocytes with either GFP (control) or PRDM16 cDNA, followed by adipogenic induction of the transduced cells. The expression of SREBP target genes was very low in undifferentiated 3T3-L1 cells, and we did not observe a significant change in response to the ectopic expression of PRDM16 (Figure 7A). However, the differentiation of the PRDM16 transduced cells was significantly reduced, both when monitored by oil red O staining (Figure 7B) and the expression of adipogenic marker genes (Figure 7C). Importantly, the expression of SREBP target genes was clearly reduced in the differentiated PRDM16-expressing cells (Figure 7A), suggesting that PRDM16 can attenuate the expression of these genes during adipogenesis, at least in vitro.

### 2.10. Endogenous PRDM16 Controls the Expression of SREBP Target Genes in 3T3-L1 Preadipocytes and hADSCs

**Figure 8:**
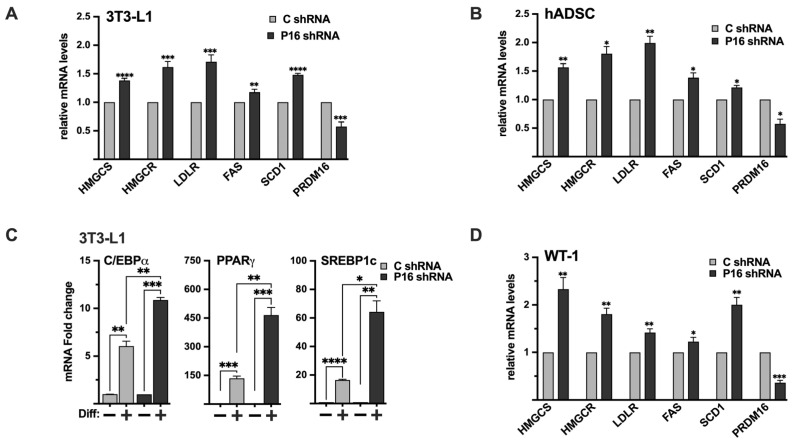
The PRDM16-mediated repression of SREBP1/2 is functional in white and brown adipocyte models. (**A**) 3T3-L1 preadipocytes were transduced with non-targeted (C) or PRDM16 (P16) shRNA. Seventy-two hours following transduction, RNA was extracted, and the expression of HMG-CoA synthase (HMGCS), HMG-CoA reductase (HMGCR), LDL receptor (LDLR), fatty acid synthase (FAS), SCD1, and PRDM16 was determined by qPCR. The relative expression of each gene in cells transduced with non-targeted shRNA was set to 1. (**B**) Human adipose-derived stem cells were transduced with non-targeted (C) or PRDM16 (P16) shRNA. Seventy-two hours following transduction, RNA was extracted, and the expression of HMG-CoA synthase (*HMGCS*), HMG-CoA reductase (HMGCR), LDL receptor (LDLR), fatty acid synthase (FAS), SCD1, and PRDM16 was determined by qPCR. The relative expression of each gene in cells transduced with non-targeted shRNA was set to 1. (**C**) 3T3-L1 preadipocytes were transduced with non-targeted (C) or PRDM16 (P16) shRNA. Ninety-six hours following transduction, cells were either left uninduced (−) or induced (+) to undergo adipocyte differentiation (Diff). Seven days after the initiation of differentiation, RNA was extracted, and the expression of C/EBPα, PPARγ, and SREBP1c was determined by qPCR. The expression of each gene in undifferentiated cells (C or P16) was set to 1. The expression of PRDM16 is shown in Figure S7. (**D**) WT-1 brown preadipocytes were transduced with non-targeted (C) or PRDM16 (P16) shRNA. Ninety-six hours following transduction, RNA was extracted, and the expression of HMG-CoA synthase (HMGCS), HMG-CoA reductase (HMGCR), LDL receptor (LDLR), fatty acid synthase (FAS), SCD1, and PRDM16 was determined by qPCR. The relative expression of each gene in cells transduced with non-targeted shRNA was set to 1. The data represent the average −/+ SEM of at least three independent experiments. *p*-values lower than 0.05 were considered statistically significant. * *p* < 0.05, ** *p* < 0.01, *** *p* < 0.001, and **** *p* < 0.0001.

Although the expression of PRDM16 mRNA was relatively low in 3T3-L1 preadipocytes, the expression of SREBP target genes was induced following shRNA-mediated knockdown of PRDM16 (Figure 8A), confirming that the functional interaction between PRDM16 and SREBP1/2 extends to this cell type. Although 3T3-L1 cells are an established cell line to study white adipogenesis, we wanted to confirm our results in primary human adipocyte progenitor cells. For this purpose, we took advantage of adipose-derived stem cells obtained from human donors. As in the 3T3-L1 preadipocytes, inactivation of PRDM16 in these cells resulted in an increased expression of several SREBP target genes involved in fatty acid and cholesterol synthesis/metabolism (Figure 8B). Interestingly, the expression levels of SREBP1a and SREBP2 were reduced in the PRDM16 knockdown ADSCs while the expression of SREBP1c was slightly increased (Figure S6). Although the increase in SREBP1c expression was statistically significant, the increase was less than 10%, suggesting that it may not be biologically relevant. Thus, we conclude that the increased expression of SREBP target genes in PRDM16 knockdown ADSCs is not the result of enhanced expression of the genes encoding SREBP1/2.

To determine if the loss of PRDM16 could affect the differentiation of preadipocytes, 3T3-L1 cells were transduced with either untargeted or PRDM16 shRNA followed by adipogenic differentiation. Interestingly, the expression of the adipogenic marker genes PPARγ, C/EBPα, and SREBP1c was induced in mature adipocytes generated from the PRDM16 knockdown preadipocytes (Figure 8C), suggesting that the loss of PRDM16 mediated repression of SREBP1/2 may enhance the differentiation of 3T3-L1 cells. The expression of PRDM16 in these cells is shown in Figure S7.

Taken together, these results demonstrate that the PRDM16-SREBP1/2 axis is functional in mouse and human preadipocytes and suggest that the lack of PRDM16-mediated repression of SREBP1/2 can promote adipogenesis in 3T3-L1 cells.

### 2.11. Inactivation of PRDM16 in Brown Preadipocytes Enhances the Expression of SREBP Target Genes

Although it is well established that SREBP1c and its target genes, including fatty acid synthase (FAS) and stearoyl-CoA desaturase (SCD1), play important roles in white adipocytes, less is known about the involvement of SREBP1/2 in brown and beige adipocytes. To explore the functional interaction between PRDM16 and SREBP1/2 in brown adipocytes, we took advantage of the WT-1 brown preadipocyte cell line. Initially, we monitored the expression of SREBP1/2, PRDM16, and their target genes in preadipocytes and following differentiation. The expression of SREBP1a, SREBP1c, SREBP2, and their target genes were relatively low in preadipocytes (Figure S8). The same was true for PRDM16, although it was expressed at higher levels than in 3T3-L1, HepG2, and MCF7 cells (based on the C_t_ values). The expression of SREBP1c and its targets FAS and SCD1 were enhanced following differentiation (Figure S8). The same was true for PRDM16 and UCP1. The mRNA levels of SREBP1a and SREBP2 were also elevated in mature WT-1 cells, as were the expression of their target genes involved in cholesterol synthesis and metabolism. Importantly, the shRNA-mediated inactivation of PRDM16 in brown preadipocytes enhanced the expression of SREBP target genes significantly (Figure 8D). The induction of HMG-CoA synthase and SCD1 was more pronounced than in 3T3-L1 and MCF7 cells, possibly because of the higher expression of PRDM16 in WT-1 preadipocytes. Given the important role of PRDM16 during brown adipogenesis, it was not possible to determine if the induction of SREBP target genes in response to PRDM16 knockdown had an impact on the differentiation of the WT-1 cells. Regardless, our results show that endogenous PRDM16 can repress SREBP target gene expression in brown preadipocytes, suggesting that the functional interaction between PRDM16 and SREBP1/2 may extend to brown adipocytes. This functional link and the mechanism(s) involved will be a focus for our future work.

## 3. Discussion

In the current manuscript, we demonstrate that PRDM16 interacts with the nuclear forms of all members of the SREBP family of transcription factors. The SREBPs interact with both zinc finger domains (ZFs) of PRDM16. These interaction domains are shared with many other transcriptional regulators that have been found to interact with PRDM16, e.g., C/EBPβ, PGC1α, MED1, and EHMT1 [48,49,53,54]. Both the N- and C-terminus of nuclear SREBP1 were required for its interaction with the ZFs in PRDM16, while deleting either domain individually had no effect on the interaction. This could indicate that SREBP1/2 and PRDM16 form multimeric complexes. Another possibility is that the deletion of both the N- and C-terminus in nuclear SREBP1 affects its structure, thereby disrupting the SREBP-PRDM16 interaction. A third possibility is that the dual interaction domains in SREBP1/2 and PRDM16 are required to form stable complexes in vivo, something that may not have been captured in our in vitro protein–protein interaction assays. The latter possibility could be addressed using sensitive in-cell protein–protein interaction techniques, such as bioluminescence resonance energy transfer (BRET) assays. Although we believe that this is the first report of a direct interaction between SREBP1/2 and PRDM16, the nuclear forms of both SREBP1 and SREBP2 were identified in a yeast two-hybrid screen for PRDM3-interacting proteins [55]. PRDM3, also known as MECOM and EVI1, displays high homology with PRDM16 and with a similar domain structure [56]. Importantly, it has been shown that PRDM3 contributes to brown adipogenesis and maintenance, especially in the absence of PRDM16 [52]. Thus, nuclear SREBP1/2 may interact with two PRDM proteins important for BAT formation and function. Exploring the functional interaction between SREBP1/2 and PRDM3 could broaden our understanding of the crosstalk between the SREBP and PRDM families of proteins.

SREBP1/2 are synthesized as membrane-bound precursor proteins that need to be cleaved in order to generate the transcriptional active forms of the proteins. In our experiments, PRDM16 behaved as a transcriptional repressor of nuclear SREBP1/2. This is illustrated by our observation that PRDM16 was able to repress the transcriptional activities of ectopically expressed nuclear SREBP1/2, and that PRDM16 failed to affect the expression of SREBP target genes in cells treated with 25-hydroxycholesterol, a potent inhibitor of SREBP1/2 activation. It will be important to identify the mechanism(s) involved in the PRDM16-mediated regulation of nuclear SREBP1/2. One possibility is that PRDM16 blocks the DNA binding of SREBP1/2. However, this is an unlikely scenario for several reasons. First, PRDM16 was able to repress the transcriptional activities of SREBP1/2 fused to the DNA binding domain of Gal4 in promoter-reporter assays that were independent of the intrinsic DNA binding activities of SREBP1/2. Secondly, we were unable to observe any effect of PRDM16 on the DNA binding of SREBP1 in our DNA binding assays. Rather, these assays suggested that PRDM16 is recruited to SREBP target promoters though its interaction with SREBP1/2. The physical and functional interactions between endogenous PRDM16 and nuclear SREBP1/2 on SREBP target promoters needs to be further explored using chromatin immunoprecipitation (ChIP) assays. A more likely possibility is that PRDM16-interacting transcriptional co-repressors are recruited to SREBP1/2 target genes as a result of the SREBP-PRDM16 interaction. Of the repressors interacting with PRDM16, CtBP1/2 are of special interest. First, CtBP1/2 does not interact with the ZFs of PRDM16 [50], suggesting that PRDM16 could interact with CtBP1/2 and SREBP1/2 simultaneously. Importantly, CtBP2 has been found to regulate the transcriptional activities of nuclear SREBP1/2, possibly in a cell type-specific manner [57,58]. CtBP2 was found to activate the expression of SREBP target genes in glioblastoma cells, while it repressed the expression of the same set of genes in liver cells. Interestingly, CtBP1/2 have been found to both bind and be controlled by NADH and fatty acids [58]. Thus, it would be interesting to explore the potential role of CtBP1/2 in the PRDM16-mediated repression of SREBP1/2. This possibility could be addressed by analyzing the recruitment of CtBP1/2 to SREBP target promoters in vivo in response to either loss or gain of PRDM16.

In fact, nuclear SREBP1/2 and PRDM16 share several interaction partners and/or regulators, including CtBP1/2, PGC1α/β, SirT1, MED1, LSD1, and C/EBPβ, suggesting that the two proteins could compete for a limited amount of these factors. The Mediator complex is a multi-protein complex that has been shown to regulate many transcription factors. Through their N-terminal transactivation domains, SREBP1/2 have been found to interact with several components of this complex, including MED14, MED15, and MED1 [27,59,60]. PRDM16 has also been shown to interact with MED1, and this interaction is important for the PRDM16-mediated control of BAT function [53,54]. Members of the C/EBP family of transcription factors play well-documented roles during adipogenesis, both white and brown/beige. C/EBPβ is involved in the expression of PRDM16 in brown adipocytes. In addition, it interacts with PRDM16 and to enhance the expression of PGC1α, thereby promoting mitochondrial biogenesis/function and the establishment of the thermogenic program. C/EBPβ is also an important regulator of SREBP1c expression and function, both in liver and during white adipogenesis [61,62]. In liver, C/EBPβ controls the insulin-dependent induction of SREBP1c mRNA [61]. Since both SREBP1/2 and C/EBPβ interact with the two ZFs in PRDM16, one possibility could be that PRDM16 represses the expression of SREBP1c by sequestering C/EBPβ, thereby reducing the expression of SREBP target genes. Alternatively, the C/EBPβ-PRDM16 complex could be recruited to the SREBP1c promoter and repress SREBP1c mRNA expression. However, although very plausible, none of these options would require a direct interaction between SREBP1/2 and PRDM16. In addition, it would not explain how PRDM16 is able to repress the transcriptional activities of ectopically expressed nuclear SREBP1/2. Regardless, further studies of the functional interactions between PRDM16, SREBP1/2, and C/EBPβ are clearly warranted, especially in adipocytes.

PRDM16 possess intrinsic methyltransferase activity and has been found to methylate lysine 9 in histone H3 (H3K9) [56,63]. H3K9 methylation is a repressive epigenetic mark associated with gene silencing and/or heterochromatin. In addition, PRDM16 has been found to interact with other proteins that either add (EHMT1) or remove (LSD1) methyl groups from histones [49,64]. Thus, it is possible that PRDM16 could control the expression of SREBP target genes by controlling epigenetic histone modifications, including methylation, at the corresponding promoters. This could be explored using modification-specific histone antibodies in ChIP experiments.

SREBP1c has been shown to support white adipogenesis in vitro, and PRDM16 is a master regulator of brown and beige adipogenesis and function. The role of PRDM16 in the browning of WAT in response to external signals is of great clinical importance. This is exemplified by the observation that the inactivation of PRDM16 in WAT and BAT resulted in the development of obesity, severe insulin resistance, and hepatic steatosis, and the investigators demonstrated that these phenotypes linked to a dramatic reduction in the browning of WAT, and thereby a significant reduction in energy expenditure [40]. We have demonstrated that PRDM16 is a repressor of SREBP1/2 in preadipocytes, and that the overexpression of PRDM16 blocks the adipogenesis and expression of SREBP target genes in 3T3-L1 cells. We have also demonstrated that the inactivation of PRDM16 enhances the expression of SREBP target genes in white and brown preadipocyte cell lines, as well as in adipose-derived human stem cells. Importantly, the expression of adipogenic marker genes was enhanced in mature adipocytes generated from PRDM16 knockdown 3T3-L1 preadipocytes. Based on our data, we propose that the PRDM16-dependent repression of SREBP1/2 could be especially important in brown/beige (pre)adipocytes and/or during the browning of WAT. This hypothesis is supported by the phenotype of transgenic mice expressing the nuclear forms of either SREBP1a or SREBP1c in adipose tissue [65,66]. Although the effects on WAT were different in these two transgenic lines, brown adipocytes in both models resembled white adipocytes and expressed high levels of classical SREBP target genes and accumulated very high levels of lipids. Importantly, the expression of UCP1, a classical BAT marker, was drastically reduced in BAT isolated from both transgenic models. Thus, the PRDM16-mediated repression of SREBP1/2 may be important to prevent lipid overload in brown/beige (pre)adipocytes and ensure that they maintain their thermogenic identity. Recent work has shown that the SREBP1c-dependent activation of fatty acid synthesis is essential to sustain BAT thermogenesis during chronic cold [37]. A similar observation was made in beige adipocytes using single-cell transcriptomics of adipose tissue from young and aged mice exposed to chronic cold [67]. The expression of both SREBP1c and its target genes involved in fatty acid synthesis was increased in beige adipocytes isolated from young mice exposed to chronic cold. Interestingly, the cold-induced beige adipogenesis and the expression of SREBP1c and its target genes were blunted in aged mice [67], suggesting that beige adipogenesis could be dependent on the reprograming of lipid metabolism. The dynamic relationship between lipid synthesis and utilization in beige and brown adipocytes may provide these fat depots with the plasticity needed to respond to metabolic/energy stress. Importantly, it suggests that this plasticity could be lost with increasing age, a condition associated with increased risk of developing metabolic disease. Thus, the functional link between PRDM16 and SREBP1/2 in adipose tissue warrants further exploration in vivo.

Based on the results reported in this manuscript, we suggest that the PRDM16-mediated repression of nuclear SREBP1/2 represents a novel mechanism to regulate lipid synthesis and metabolism. We propose that the functional interactions between these transcriptional regulators could impact adipose biology, both WAT and BAT. The SREBP2-LDL receptor axis is already a well-established target for cholesterol-lowering therapeutics in cardiovascular disease, and PRDM16 is a very attractive target for obesity and T2D. However, PRDM16 regulates other important biological processes beyond adipogenesis, including in the brain, intestine, and cancer. Thus, the different interactions involving PRDM16 have emerged as potential targets in metabolic disease. It will be interesting to see if the PRDM16-SREBP1/2 axis is a valid therapeutic target in metabolic disease.

## 4. Materials and Methods

### 4.1. Cell Culture and Treatments

MCF7 (HTB-22), HepG2-C3A (CRL-10741), HEK293 (CRL-1573), and 3T3-L1 (MBX.CRL-3242) cells were obtained from American Type Cell Culture Collection (ATCC), and human adipose-derived stem cells (R7788110) were obtained from Thermo Fisher Scientific (Waltham, MA, USA) and were cultivated in MesenPRO RS medium (Thermo Fisher Scientific, 12746012). WT-1 mouse brown preadipocytes cells (SCC255) were obtained from Merck Millipore (Burlington, MA, USA). All other cell culture media, supplements, and reagents were from Thermo Fisher Scientific. HepG2-C3A cells were cultured in MEM media supplemented with 10% FBS, non-essential amino acids, sodium pyruvate, Glutamax and antibiotic-antimycotic, and MCF7, HEK293, and 3T3-L1 cells were cultured in DMEM media supplemented with 10% FBS in addition to the supplements mentioned above. Where indicated, HepG2 and MCF7 cells were grown in media in which FBS was replaced by lipoprotein-deficient sera (Merck Millipore) to promote activation of SREBP1/2.

### 4.2. Adipocyte Differentiation

3T3-L1 preadipocytes were allowed to reach confluency, at which point the media was changed. Forty-eight hours after reaching confluency, the media was changed to adipocyte differentiation media, which was composed of regular growth media supplemented with 0.5 mM isobutyl-1-methylxanthine (IBMX), 1 μM dexamethasone, and 4 μg/mL human insulin. Cells were left in differentiation media for 48 h, after which the media was changed to regular media supplemented with insulin (4 μg/mL). The experiments were stopped 7 days after the addition of differentiation media. For the differentiation of WT-1, cells were incubated with an induction medium (DMEM high glucose, 2% FBS, 20 nM insulin, 1 nM triiodo-L-thyronine (T3), 0.125 mM indomethacin, 5 μM dexamethasone, 0.5 mM IBMX, and 1% penicillin/streptomycin) for 48 h, prepared fresh and sterile-filtered on the day of use. After 48 h, the induction medium was changed to differentiation medium (DMEM high glucose, 2% FBS, 20 nM insulin, 1 nM T3, and 1% penicillin/streptomycin). Differentiation medium was refreshed every other day, and the experiments were stopped 7 days post induction.

### 4.3. Plasmid DNA

The lentiviral shRNA constructs targeting human PRDM16 (RHS3979-201751161-TRCN0000020044, RHS3979-201751162-TRCN0000045, and RHS3979-201751163-TRCN0000046) were purchased from Horizon Discovery (Cambridge, UK). The corresponding constructs targeting mouse PRDM16 (VB900137-6002rva and VB900137-6003bzy) and the lentiviral expression vector for human PRDM16 (NM-022114-4; VB900131-6471kkm) were purchased from VectorBuilder (Chicago, IL, USA). pCaggs PRDM16-F/H was kindly provided by Thomas Jenuwein [56]. GST-PRDM16-1-223 (#53346), GST-PRDM16-224-454 (#53347), GST-PRDM16-455-680 (#53348), GST-PRDM16-681-880 (#53349), GST-PRDM16-880-1038 (#53350), and GST-PRDM16-1039-1176 (#53351) constructs were kindly provided by Bruce Spiegelman [50]. pGL2-SYNSRE-luciferase (#60444) and of pGL2-SYNSREΔSRE-luciferase (#60490) promoter-reporter constructs were kindly provided by Timothy Osborne [68]. pGL4-LDLR-luciferase, pGL4-LDLRΔSRE-luciferase, pGL4-FAS-luciferase, and pGL3-G1E1B-luciferase have been described previously [25,69,70]. The expression vectors for FLAG and MYC-tagged nSREBP1s (1a, 1c, and 2) and the deletion mutants of FLAG-nSREBP1a (FLAG-SREBP1a-ΔTAD (amino acids residues 90–490)), FLAG-SREBP1a-ΔC (amino acid residues 2–417), and FLAG-SREBP1aΔTADΔC have been described previously [69,71]. The Gal-4 DNA binding domain (amino acid residues 1–147) cloned into pcDNA3 was used to develop Gal4-SREBP1/2 (1a, 1c, or 2) and Gal4-TAD (1a, 1c, or 2), as described previously [72,73].

### 4.4. Lentivirus Production and Transduction

HEK293 cells grown in 10 cm dishes were used to produce all lentiviruses. Twelve μg of lentiviral DNA was co-transfected with 15 μL Trans-Lentiviral shRNA Packaging Kit (Horizon Discovery, TLP5912) by the calcium phosphate precipitation transfection method. Forty-eight hours after transfection, media was collected and filtered through 0.45 μm syringe filters, and the viruses were stored in aliquots at −80 °C. Target cells were transduced in regular media containing 8 μg/mL polybrene for 16 h, followed by 3–4 days of puromycin selection (2 μg/mL). The viruses expressing shRNA targeting human and mouse PRDM16 were produced and used as pools consisting of three and two shRNAs, respectively (see Section 4.3).

### 4.5. Antibodies and Reagents

Antibodies against PRDM16 (16212) and SCD1 (2438S) were purchased from Cell Signaling Technology (Danvers, MA, USA). Antibodies against HMG-CoA synthase (sc-271543), SREBP1 (sc-8984 and sc-13551), GST (sc-138), Myc (sc-40), and HA (sc-805) were purchased from Santa Cruz Biotechnology (Dallas, TX, USA), and the SREBP2 antibody (AF7119) was purchased from R&D Systems (Minneapolis, MN, USA). Anti-FLAG (F3165) antibody was obtained from Merck Millipore. The LDL receptor antibody (PA5-22976) was purchased from Thermo Fisher Scientific, and the β-actin antibody (A5441) was purchased from Merck Millipore. Horseradish peroxidase (HRP)-conjugated anti-rabbit IgG (G21234) and anti-mouse IgG (62-6520) antibodies were purchased from Thermo Fisher Scientific. Horseradish peroxidase (HRP)-conjugated anti-goat IgG (HAF019) antibody was purchased from R&D Systems. Chemicals were obtained from Merck Millipore, unless otherwise indicated.

### 4.6. Cell Lysis and Immunoblotting

Cells were lysed in buffer A (50 mM HEPES (pH 7.2), 150 mM NaCl, 1 mM EDTA, 20 mM NaF, 2 mM sodium orthovanadate, 10 mM β-glycerophosphate, 1% (*w*/*v*) Triton X-100, 10% (*w*/*v*) glycerol, 1 mM phenylmethylsulfonyl fluoride (PMSF), 10 mM sodium butyrate, 1% aprotinin, 0.1% sodium dodecyl sulfate (SDS), and 0.5% sodium deoxycholate (DOC)) and cleared by centrifugation [73]. SDS and DOC were omitted from the lysis buffer for protein–protein and protein–DNA interaction assays. Proteins were resolved by SDS–PAGE (4–12% Bis-Tris; Invitrogen) and transferred to nitrocellulose membranes (Cytiva, Marlborough, MA, USA). Membranes were blocked in 5% BSA in PBS containing 0.05% Triton X-100, probed with primary and HRP-conjugated secondary antibodies, and visualized by chemiluminescence on an iBright CL1500 (Thermo Fisher Scientific).

### 4.7. Protein Purification

Cultures of *E. coli* (BL21) transformed with expression vectors for 6xHis-nSREBP1a or GST-PRDM16 were induced with IPTG (0.75 mM) and incubated overnight at room temperature with shaking to allow protein expression. Cells were harvested by centrifugation, and the cell pellets were resuspended in 20 mL PBS (ice-cold) containing protease inhibitors (PMSF and aprotinin) and sonicated on ice. Following sonication, Triton-X-100 was added to a final concentration of 1%, and the suspension was kept in an end-over-end mixer for 30 min at 4 °C. The solubilized material was centrifuged at 10,000× *g* rpm for 20 min at 4 °C, and the supernatant was collected in new tubes. Clarified lysates were used to purify the His- and GST-tagged proteins using Ni-NTA (Merck Millipore, P661) and glutathione Sepharose (Cytiva, 17-5132-01), respectively, employing standard protocols [72]. The GST-tagged proteins were either retained on the glutathione beads for GST pulldown assays or eluted with an excess of glutathione for electromobility shift assays. The purified 6xHis-SREBP1a protein was eluted from the Ni-NTA beads with imidazole. All eluted proteins were dialyzed against PBS containing 20% glycerol and protease inhibitors (PMSF and aprotinin), aliquoted and stored at −80 °C.

### 4.8. GST Pulldown and Co-Immunoprecipitation Assays

GST pulldown assays were performed using GST-tagged PRDM16 fragments as bait and Myc-tagged nuclear SREBP1/2 (nSREBP1a, nSREBP1c, nSREBP2) expressed in HEK293 cells as prey. HEK293 cells were transfected by calcium phosphate precipitation with plasmids encoding Myc-tagged nuclear SREBP1/2, either wild-type or the indicated deletion mutants. Cell lysates were prepared in buffer A without SDS and DOC and pre-cleared using glutathione beads. The pre-cleared lysates (125 µL per reaction) were incubated with immobilized GST-PRDM16 fragments on glutathione beads for 1 h at 4 °C with rotation. Beads were collected by centrifugation and washed three times in buffer A, once in 0.5M NaCl, followed by a final wash in buffer A. The pulled-down material and inputs were resolved by SDS–PAGE and processed for Western blotting. Co-immunoprecipitation assays were performed as described [25,70]. In brief, HEK293 cells were transfected with expression vectors for Myc-tagged nuclear SREBP1c or SREBP2 in the absence or presence of HA-tagged PRDM16. Forty-eight hours after transfection, cell lysates were prepared in buffer A without SDS and DOC, and pre-cleared with protein A agarose beads (Millipore, P9424). The pre-cleared lysates were mixed with anti-HA antibodies (1 μg) and placed in an end-over-end mixer for three hours at 4 °C, followed by the addition of protein A agarose beads. The protein A agarose beads were collected by centrifugation and washed three times in buffer A, once in 0.5 M NaCl, followed by a final wash in buffer A. The immunoprecipitated proteins were resolved by SDS–PAGE and processed for Western blotting.

### 4.9. DNA Pulldown Assay

DNA pulldown assays were performed as described previously [74]. Biotin-labeled DNA probes corresponding to wild-type or the ΔSRE version of the LDL receptor promoter were generated by PCR using biotin-labeled primers and the appropriate pGL4-LDLR templates. The primer sequences were as follows: Biotin-CTA GCA AAA TAG GCT GTC CC and CTT TAT GTT TTT GGC GTC TTC CA. DNAP reactions (typically 100 µL) were assembled in 20 mM Tris-HCl pH 7.5, 50 mM NaCl, 20% glycerol, 1 μg sheared salmon sperm DNA (non-competitive DNA), 1 mM MgCl2, 1 mM dithiothreitol, and 0.5 μg bovine serum albumin (BSA). Biotin-DNA probes (100 ng) were incubated with purified GST-PRDM16 fragments (ZF1, aa 224–454; or ZF2, aa 881–1038) in the absence or presence of 6xHis-SREBP1a at 4 °C for 90 min. Finally, the DNA probes were captured with streptavidin magnetic beads (Thermo Fisher Scientific, 8816), washed four times with cold binding buffer, and eluted in Laemmli buffer. The captured proteins were separated on SDS-PAGE gels, transferred to nitrocellulose membranes, and immunoblotted with anti-His and anti-GST antibodies.

### 4.10. Electromobility Shift Assays

Electromobility shift assays were performed as described earlier [75], but with the use of unlabeled DNA probes. In brief, unlabeled DNA probes corresponding to wild-type or the ΔSRE version of the LDL receptor promoter were generated by PCR using the appropriate pGL4-LDLR templates. The primer sequences were as follows: CTA GCA AAA TAG GCT GTC CC and CTT TAT GTT TTT GGC GTC TTC CA. The 10x binding buffer contains 200 mM Tris-HCl pH 7.5 and 500 mM NaCl. The final reaction mixture contained 2 μL 10x binding buffer, 20% glycerol, 1 μg sheared salmon sperm DNA (non-competitive DNA), 1 mM MgCl2, 1 mM dithiothreitol, and 0.5 μg bovine serum albumin (BSA). GST-ZF1 or GST-ZF2 were incubated with 500 ng of DNA probe in the absence or presence of 6xHis-SREBP1a. Where indicated, anti-His or anti-GST antibodies (0.5 μg) were added to the reaction. In the experiment illustrated in Figure 5D, the recombinant proteins were replaced with nuclear extracts from HEK293 cells transfected with either empty vector, nuclear SREBP1a, or PRDM16. The reactions were separated on 4% polyacrylamide gels with 0.5x TBE buffer, stained with SYBR Safe, and visualized on an iBright CL1500 Imaging System (Thermo Fisher Scientific).

### 4.11. Luciferase and β-Galactosidase Assays

Promoter-reporter assays were performed as described previously [26,69,71]. In brief, HepG2 cells were transiently transfected with the indicated promoter-reporter genes in the absence or presence of the indicated expression and/or shRNA vectors. Luciferase activities were determined in duplicate samples as described by the manufacturer (Promega, Madison, WI, USA). Cells were also transfected with the β-galactosidase gene as an internal control for transfection efficiency. Luciferase values (relative light units, RLUs) were calculated by dividing the luciferase activity by the β-galactosidase activity. The data represent the average −/+ SEM of at least three independent experiments performed in duplicates.

### 4.12. RNA Extraction and qPCR

RNA was extracted using GeneJet RNA Purification Kit (Thermo Fisher Scientific). cDNA was generated using Applied Biosystems High-Capacity cDNA Reverse Transcription Kit ((Thermo Fisher Scientific). For qPCR, PowerUp SYBR Green Master Mix was used ((Thermo Fisher Scientific), using cyclophilin, hypoxanthine phosphoribosyltransferase (HPRT1), RPLP0, and actin as reference/housekeeping genes. HPRT1 and cyclophilin were used for MCF7 cells and ADSCs, HPRT1, cyclophilin and actin were used for 3T3-L1, and RPLP0 was used for WT-1 cells. The human and mouse primer sequences used to amplify target genes are provided in Tables S1 and S2, respectively.

### 4.13. Oil Red O Staining

Oil Red O staining of lipids was performed using a well-established protocol. Briefly, cells were washed twice in PBS, then fixed in 4% (*v*/*v*) formaldehyde for 30 min at room temperature. The fixed cells were washed three times with PBS and once with 60% isopropanol. The fixed cells were treated with freshly prepared and filtered Oil Red O staining solution in 60% isopropanol for 60 min, followed by extensive washes with water. The stained cells were left immersed in water until imaging.

### 4.14. LipidTox Staining of Neutral Lipids

LipidTOX Green (H34475) was used according to the manufacturer’s instructions (Thermo Fisher Scientific) [26]. Briefly, cells grown in 12-well plates were fixed in 3.5% (*v*/*v*) formaldehyde and washed extensively with PBS. The stain was used at a 1:1000 dilution in PBS. Cells were stained for 2 h and kept in PBS at 4 °C until imaging. At least 5 random fields/well were captured on an inverted microscope (Olympus IX73, Center Valley, PA, USA) using identical settings and exposure times. Representative images are displayed in the panels. The fluorescence in each image was quantified in Fiji and corrected for cell numbers. The mean fluorescence intensities −/+ SD across all images within each experimental group are provided in the figures.

### 4.15. LDL Uptake Assays

The pHrodo Red-LDL (Thermo Fisher Scientific, L34356) is dimly fluorescent at neutral pH but becomes brightly fluorescent after endocytosis. The cells were rinsed with PBS and incubated in media containing lipoprotein-deficient sera for 16 h. pHrodo Red-LDL was added to a final concentration of 8 μg/mL and followed by a 4-h incubation at 37 °C in a cell culture incubator. The cells were washed twice with PBS containing BSA (0.3%), and the pHrodo Red LDL-stained cells were imaged in the rhodamine red channel using appropriate filter sets. At least 5 random fields/well were captured on an inverted microscope (Olympus IX73) using identical settings and exposure times. Representative images are displayed in the panels. The fluorescence in each image was quantified in Fiji and corrected for cell numbers. The mean fluorescence intensities −/+ SD across all images within each experimental group are provided in the figures.

### 4.16. Statistical Analysis

Statistical analyses were performed using GraphPad Prism version 10 (GraphPad Software). For comparisons involving more than two groups of a single experimental factor, Welch’s one-way ANOVA (Brown–Forsythe and Welch ANOVA test) was applied as this approach does not assume equal variances. When the ANOVA indicated significance, Dunnett’s T3 multiple comparisons test was used for post-hoc pairwise group comparisons. For direct comparisons between two groups, Welch’s *t*-test was employed. Data are presented as mean ± SEM, unless otherwise indicated. A *p*-value < 0.05 was considered statistically significant.

## Data Availability

The original contributions presented in this study are included in the article/Supplementary Material. Further inquiries can be directed to the corresponding authors.

## Supplementary Materials

The following supporting information can be downloaded at: https://www.mdpi.com/article/10.3390/ijms262110246/s1.

## Abbreviations

The following abbreviations are used in this manuscript:

SREBP	Sterol Regulatory Element-Binding Protein
PRDM	PRDI-BF1 and RIZ Homology Domain Containing
SCAP	SREBP Cleavage Activating Protein
Insig	Insulin-induced Gene
PPAR	Peroxisome Proliferator–Activated Receptor
PGC1	Peroxisome Proliferator-Activated Receptor Gamma Coactivator 1
C/EBP	CCAAT-Enhancer-Binding Protein
FAS	Fatty acid synthase
SCD1	Stearoyl-CoA 9-desaturase
LDL	Low-density Lipoprotein
LDLR	Low-density Lipoprotein Receptor
HMGCS	HMG-CoA Synthase
HMGCR	HMG-CoA Reductase
25-HC	25-Hydroxycholesterol
ER	Endoplasmic Reticulum
GFP	Green Fluorescence Protein
GST	Glutathione S-Transferase
SRE	Sterol Regulatory Element
shRNA	Short Hairpin RNA
qPCR	Quantitative Polymerase Chain Reaction
LDM	Lipoprotein-Deficient Media
ADSC	Adipose-Derived Stem Cells
ZF	Zinc Finger
TAD	Transactivation Domain
EMSA	Electromobility Shift Assay
mRNA	Messenger RNA
PCR	Polymerase Chain Reaction
UCP1	Uncoupling Protein 1
EHMT1	Euchromatic Histone Methyltransferase 1
MED	Mediator
BAT	Brown Adipose Tissue
WAT	White Adipose Tissue
CtBP1/2	C-Terminal-Binding Protein 1/2
LSD1	Lysine-Specific Histone Demethylase 1
T2D	Type-2 Diabetes
