# Supplementary material for: The Functional Interaction Between PRDM16 and the SREBP Pathway Controls Lipid Metabolism
Source: Int J Mol Sci. 2025 Oct 22;26(21):10246. doi: 10.3390/ijms262110246 (PMC12609382; doi:10.3390/ijms262110246)
Supplement: Supplementary file 1 [file ijms-26-10246-s001.zip › ijms-3875412-supplementary.pdf]

## SUPPLEMENTARY FIGURES

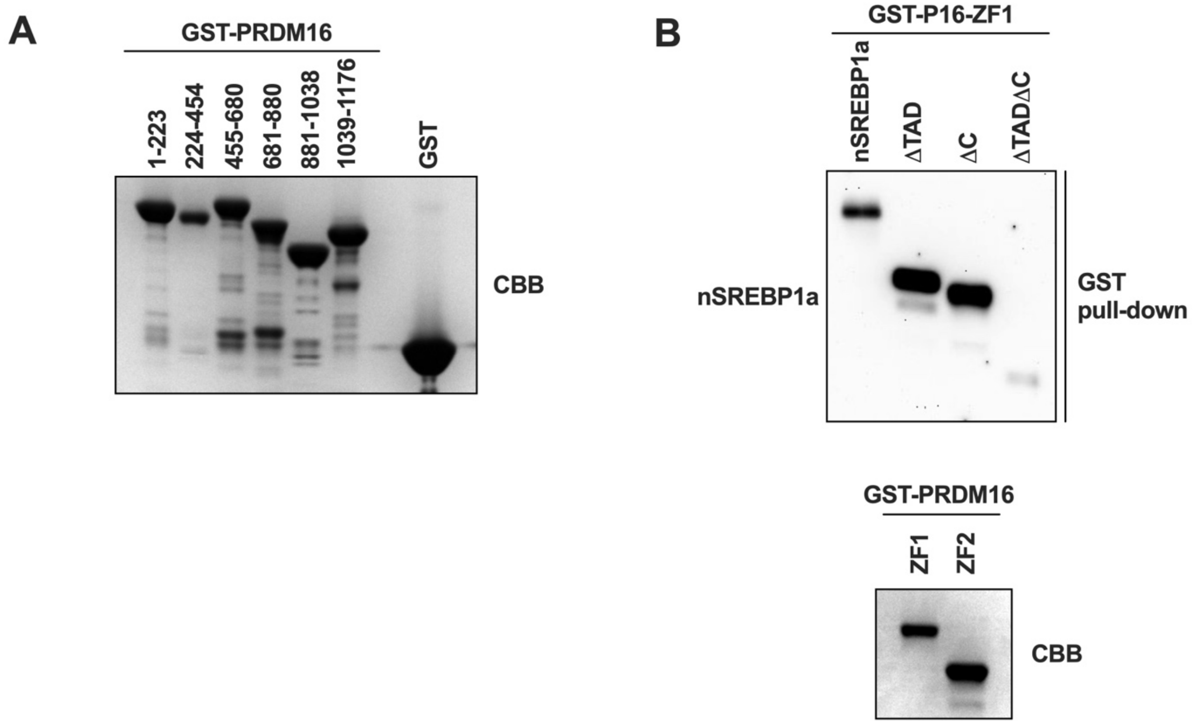


Figure S1. **Nuclear SREBP1/2 interacts with the zinc fingers in PRDM16**. (**A**) Coomassie-stained SDS-PAGE gel illustrating the purity and relative levels of the GST-PRDM16 fragments used in the GST pulldown assays in Figure 4C. (**B**) HEK293 cells were transfected with expression vectors encoding full-length nuclear SREBP1a (nSREBP1a), or the indicated deletion mutants, i.e., deletion of the N-terminal transactivation domain (ΔTAD), the C-terminal regulatory domain (ΔC) or both domains (ΔTADΔC). Whole-cell lysates were used in GST pulldown assays using GST-ZF as bait. The pulldown material was analyzed by Western blotting. In the lower panel, a Coomassie stained SDS-PAGE gel illustrating the purity and relative amount of GST-PRDM16-ZF1 (ZF1) and GST-PRDM16-ZF2 (ZF2) used in the GST pulldown assays in Figure S1B and Figure 4D, respectively.

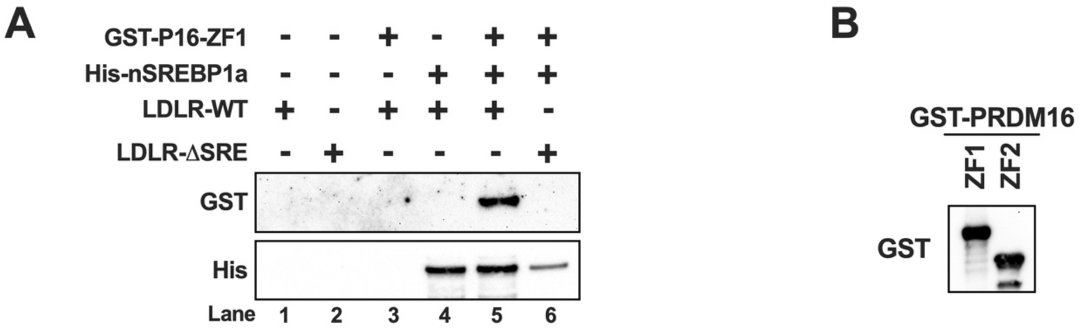


Figure S2. **The recruitment of PRDM16 to SREBP target promoters is dependent on its interaction with nuclear SREBP.** (**A**) GST-tagged PRDM16-ZF1 (*GST-P16-ZF1*) and 6xHis-tagged nuclear SREBP1a (*His-nSREBP1a*) were used in DNA-precipitation assays using two different biotin-labeled LDL receptor promoter probes, either wild-type (*LDLR-WT*) or the corresponding SREBP binding site deletion (*LDLR-ΔSRE*). The recombinant proteins were mixed with the promoter probes, either individually or together, and the DNA-protein complexes were captured on streptavidin-coated magnetic beads. Following extensive washing, the captured proteins were separated on SDS-PAGE gels and the amounts of ZF1 (*GST*) and *nSREBP1a* (His) were analyzed by Western blotting. (**B**) Western blot of GST-tagged zinc finger 1 (ZF1) and 2 (ZF2) used in the DNAP assays shown in Figure S2A and Figure 5A, respectively.

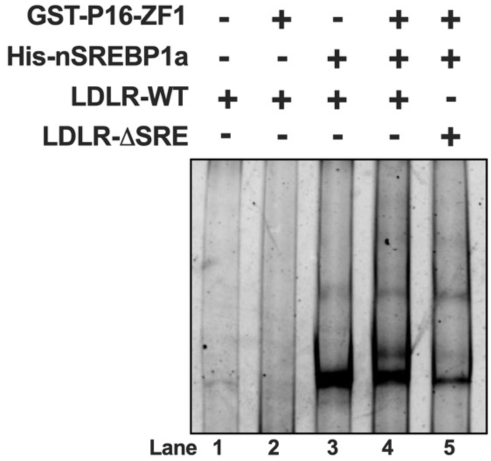


Figure S3. **Nuclear SREBP recruits PRDM16 to target promoters *in vitro***. GST-tagged PRDM16-ZF1 (*GST-P16-ZF1*) and 6xHis-tagged nuclear SREBP1a (*His-nSREBP1a*) were used in electromobility shift assays using two different unlabeled LDL receptor promoter probes, either wild-type (*LDLR-WT*) or the corresponding SREBP-binding site deletion (*LDLR-ΔSRE*). The recombinant proteins were mixed with the promoter probes, either individually or together, and the DNA-protein complexes were resolved on native PAGE gels and visualized with SYBR Safe. Related to Figure 5B.

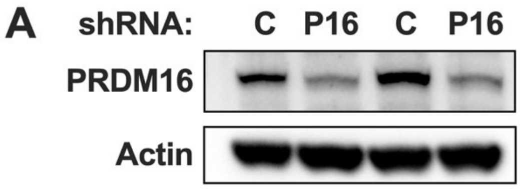


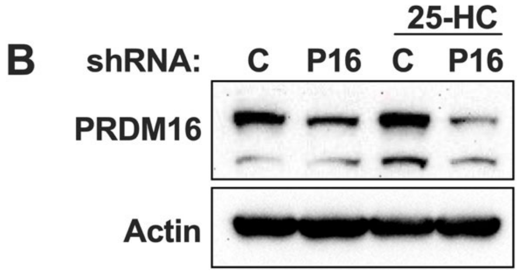


Figure S4. **Confirmation of the efficiency of PRDM16-targeted shRNA in HepG2 and MCF7 cells**. (**A**) HepG2 cells were transduced with non-targeted (*C*) or PRDM16 (*P16*) shRNA. Ninety-six hours after transduction, whole-cell lysates were prepared and separated on SDS-PAGE gels. The levels of PRDM16 and actin (loading control) were analyzed by Western blotting. The results of two separate experiments are illustrated. Related to Figures 1-3. (**B**) MCF7 cells were transduced with non-targeted (*C*) or PRDM16 (*P16*) shRNA. Seventy-two hours after transduction, the media was changed to lipoprotein-deficient media, which was supplemented with 25-hydroxycholesterol (*25-HC*) where indicated. Ninety-six hours after transduction, whole-cell lysates were prepared and separated on SDS-PAGE gels. The levels of PRDM16 and actin (loading control) were analyzed by Western blotting. Related to Figure 6.

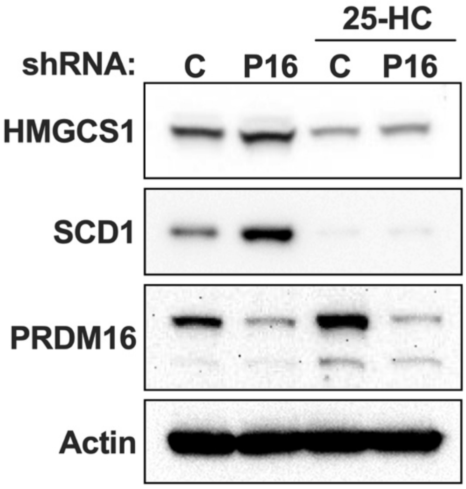


Figure S5. **Inactivation of PRDM16 enhances the expression of HMG-Co synthase and SCD1 protein.** MCF7 cells were transduced with non-targeted (*C*) or PRDM16 (*P16*) shRNA. Seventy-two hours after transduction, the media was changed to lipoprotein-deficient media, which was supplemented with 25-hydroxycholesterol (*25-HC*) where indicated. Ninety-six hours after transduction, whole-cell lysates were generated and resolved on SDS-PAGE gels. The levels of HMG-CoA synthase (*HMGCS*), SCD1, PRDM16, and actin (loading control) were analyzed by Western blotting. Related to Figure 6A.

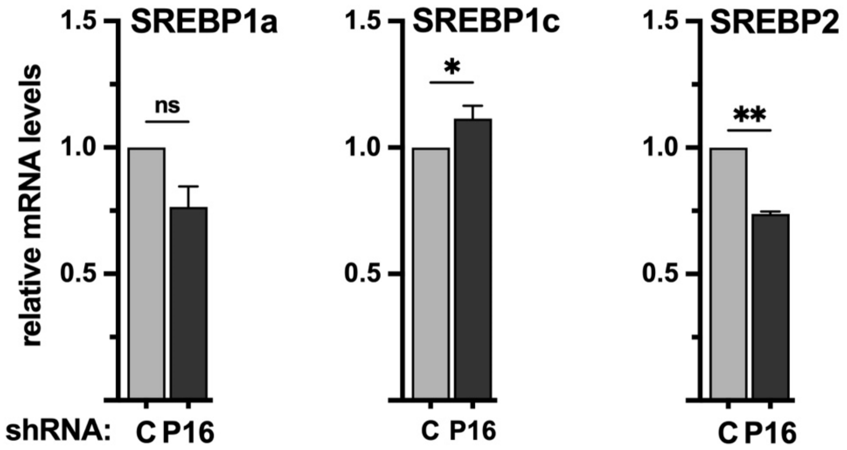


Figure S6. **The expression of SREBP1/2 is not induced in PRDM16 knockdown human ADSCs**. Human adipose-derived stem cells were transduced with non-targeted (*C*) or PRDM16 (*P16*) shRNA. Seventy-two hours following transduction, RNA was extracted and the expression of SREBP1a, SREBP1c, and SREBP2 was determined by qPCR. The relative expression of each gene in cells transduced with non-targeted shRNA was set to 1. The data represent the average −/+ SEM of at least three independent experiments. *P < 0 .05, and **P < 0 .01. *ns*, not significant. Related to Figure 8B.

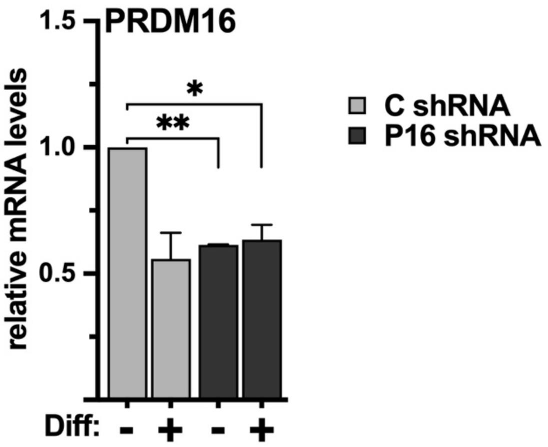


Figure S7. **Confirmation of PRDM16 knockdown in differentiated 3T3-L1 cells**. 3T3-L1 preadipocytes were transduced with non-targeted (*C*) or PRDM16 (*P16*) shRNA. Ninety-six hours following transduction, were either left uninduced (-) or induced (+) to undergo adipocyte differentiation (*Diff*). Seven days after the initiation of differentiation, RNA was extracted and the expression of PRDM16 was determined by qPCR. The expression of PRDM16 in uninduced cells transduced with non-targeted shRNA was set to 1. P-values lower than 0.05 were considered statistically significant. The data represent the average −/+ SEM of at least three independent experiments. *P < 0 .05, and **P < 0 .01. Related to Figure 8C.

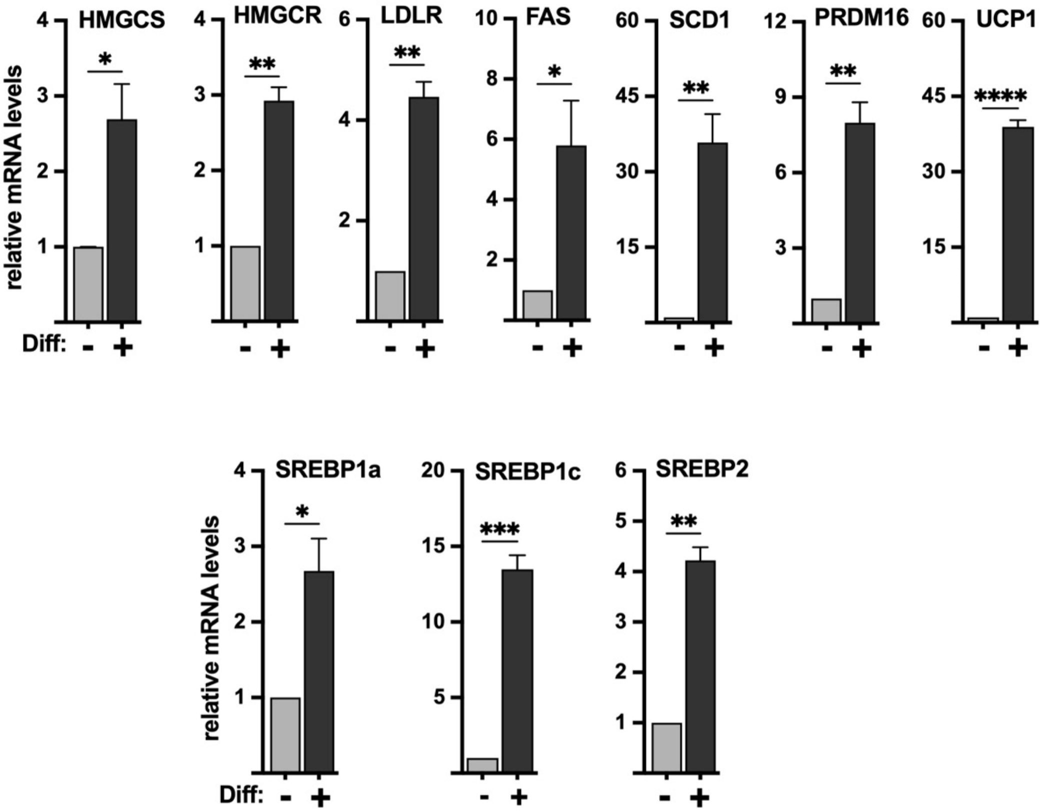


Figure S8. **SREBP1c and genes involved in fatty acid and cholesterol synthesis are induced during brown adipogenesis**. WT-1 brown preadipocytes were either left uninduced (-) or induced (+) to undergo adipocyte differentiation (*Diff*). Seven days after the initiation of differentiation, RNA was extracted and the expression of fatty acid synthase (FAS), SCD1, SREBP1c, and PRDM16 was determined by qPCR. The relative expression of each gene in uninduced cells was set to 1. The data represent the average −/+ SEM of at least three independent experiments P-values lower than 0.05 were considered statistically significant. *P < 0 .05, **P < 0 .01, ***P < 0 .001, and ****P < 0.0001. Related to Figure 8D.

**Table S1: Human qPCR Primers.**

| Gene | Primer-F | Primer-R |
|---|---|---|
| hPRDM16 | ATC AGC CAA TCT CAC CAG AC | CTG GAG GTT CGA AGA GAT GC |
| hHMGCS | GAA ACA GTG ACA GAC CTG GAG | AGC AAG CTT CTG CAT TCA AAG |
| hHMGCR | GTT TAC CCT CGA TGC TCT TGT | CTG ACA TGC AGC CAA AGC |
| hSCD1 | CTC TTT CTG ATC ATT GCC AAC AC | GGA ATT ATG AGG ATC AGC ATG TG |
| hFAS | GAA ACT GCA GGA GCT GTC | CAC GGA GTT GAG CCG CAT |
| hLDLR | CAA TGT CTC ACC AAG CTC TG | TCT GTC TCG AGG GGT AGC TG |
| hSREBP1a | TCA GCG AGG CGG CTT TGG AGC AG | CAT GTC TTC GAT GTC GGT CAG |
| hSREBP1c | GGA GGG GTA GGG CCA ACG GCC T | CAT GTC TTC GAA AGT GCA ATC C |
| hSREBP2 | CTG AAG CTG GCA AAT CAA AAG | TCA TCC AAT AGA GGG CTT CCT |
| hHPRT1 | TGA CAC TGG CAA AAC AAT GCA | GGT CCT TTT CAC CAG CCA AGC T |
| hCyclophilin | GCA GAC AAG GTC CCA AAG ACA G | CAC CCT GAC ACA TAA ACC CTG G |

**Table S2: Mouse qPCR Primers.**

| Gene | Primer-F | Primer-R |
|---|---|---|
| mHMGCS | CTG CTA TTC TGT CTA CCG CAA | TGA GTG AAA GAT CAT GAA GCC A |
| mHMGCR | CTT GTG GAA TGC CTT GTG ATT G | AGC CGA AGC AGC ACA TGA T |
| mSCD1 | GCG ATA CAC TCT GGT GCT CA | CCC AGG GAA ACC AGG ATA TT |
| mFAS | GCT GCG GAA ACT TCA GGA AAT | AGA GAC GTG TCA CTC CTG GAC TT |
| mLDLR | AGG CTG TGG GCT CCA TAG G | TGC GGT CCA GGG TCA TCT |
| mSREBP1a | GGC CGA GAT GTG CGA ACT | TTG TTG ATG AGC TGG AGC ATG |
| mSREBP1c | GGA GCC ATG GAT TGC ACA TT | CAG GAA GGC TTC CAG AGA GG |
| mSREBP2 | GCG TTC TGG AGA CCA TGG A | ACA AAG TTG CTC TGA AAA CAA ATC A |
| mPPAR-γ | CCA GAG CAT GGT GCC TTC GCT | CAG CAA CCA TTG GGT CAG CTC |
| mC/EBP-α | AAT GGC AGT GTG CAC GTC TA | CCC CAG CCG TTA GTG AAG AG |
| mAdipsin | CCT GAA CCC TAC AAG CGA TG | GGT TCC ACT TCT TTG TCC TCG |
| mActin | GGC TGT ATT CCC CTC CAT CG | CCA GTT GGT AAC AA TCC ATG T |
| mCyclophilin | TGG AGA GCA CCA AGA CAG ACA | TGC CGG AGT CGA CAA TGA T |
| mRPLP0 | TTTGGGCATCACCACGAAAA | GGACACCCTCCAGAAAGCGA |
| mUCP1 | CTTTGCCTCACTCAGGATTGG | ACTGCCACACCTCCAGTCATT |
